# Epicardial adipose tissue radiomics predicts VR and MACE after AMI: a prospective cohort study

**DOI:** 10.3389/fendo.2026.1781007

**Published:** 2026-05-18

**Authors:** Zeyan Liu, Yanfang Yang, Xiaodong Pan, Min Yang, Ye Zhang

**Affiliations:** 1 Emergency Internal Medicine Department, The Second Affiliated Hospital of Anhui Medical University, Hefei, Anhui, China; 2 Chest Pain Center (CPC), The Second Affiliated Hospital of Anhui Medical University, Hefei, Anhui, China; 3 Imaging Medicine Department, The Second Affiliated Hospital of Anhui Medical University, Hefei, Anhui, China; 4 Intensive Care Medicine Department II, The Second Affiliated Hospital of Anhui Medical University, Hefei, Anhui, China; 5 Department of Anesthesiology and Perioperative Medicine, The Second Affiliated Hospital of Anhui Medical University, Hefei, Anhui, China

**Keywords:** epicardial adipose tissue, machine learning, myocardial infarction, radiomics, ventricular remodeling

## Abstract

**Background:**

Epicardial adipose tissue (EAT) has been implicated in adverse cardiac remodeling after acute myocardial infarction (AMI), but its imaging-based characterization remains limited. This study was designed to develop and validate EAT radiomics–based models for predicting ventricular remodeling (VR) and major adverse cardiovascular events (MACE).

**Materials and methods:**

A single-center prospective cohort study enrolled 206 AMI patients at the Second Affiliated Hospital of Anhui Medical University between January 2022 and June 2023. EAT radiomics features were extracted from cardiac CT using Pyradiomics, and predictive models were constructed with machine learning algorithms. Statistical analyses were performed using R, Python, and SPSS, with significance set at P < 0.05.

**Results:**

During 12-month follow-up, 29.6% developed VR and 19.9% experienced MACE. EAT volume was independently associated with both outcomes (P < 0.001). Radiomics-based Model 2 showed superior predictive performance compared with Model 1, with higher AUC and C-index values across validation folds. Experimental studies demonstrated that EAT aggravated myocardial injury, fibrosis, and apoptosis, which were partially attenuated by IL-6 neutralization.

**Conclusion:**

Integrating EAT radiomics with clinical parameters improves prediction of VR and MACE after AMI and is supported by experimental evidence linking EAT to post-infarction remodeling.

## 1 Introduction

Schemic heart disease (IHD) remains the leading cause of morbidity and mortality worldwide, with acute myocardial infarction (AMI) representing its most severe clinical manifestation (1). Despite substantial advances in reperfusion therapy and acute clinical management, the long-term prognosis of patients after AMI remains unsatisfactory. Established risk factors for AMI include hypertension, diabetes mellitus, dyslipidemia, smoking, and obesity, with growing evidence highlighting the contribution of metabolic dysfunction and visceral adiposity to disease progression (2). Ventricular remodeling (VR), characterized by progressive ventricular dilation and functional deterioration, is a key pathological process driving adverse outcomes and is strongly associated with major adverse cardiovascular events (MACE), including recurrent myocardial infarction, heart failure, malignant arrhythmias, and death (3).The development of VR is a dynamic and multifactorial process involving inflammation, oxidative stress, neurohormonal activation, and extracellular matrix remodeling (4). Although VR is commonly defined clinically as a ≥20% increase in left ventricular end-diastolic volume (LVEDV), this definition relies on delayed imaging assessment and therefore lacks value for early risk stratification (5, 6). Moreover, traditional predictors based on single clinical or imaging parameters are insufficient to capture the complex and heterogeneous nature of post-infarction remodeling, underscoring the need for more integrative and sensitive predictive strategies (7). Importantly, early identification of patients at high risk of VR remains challenging, as subclinical pathological changes often precede overt structural alterations and may not be detected by conventional approaches (8).

Epicardial adipose tissue (EAT), a unique visceral fat depot located between the myocardium and the visceral pericardium, has recently attracted increasing attention due to its active role in cardiovascular pathophysiology (9).Unlike other adipose tissues, EAT shares a common microcirculation with the myocardium and lacks a separating fascia, enabling direct paracrine and vasocrine interactions (10). Under pathological conditions, excessive EAT accumulation is associated with increased secretion of pro-inflammatory cytokines, adipokines, and free fatty acids, which can promote myocardial inflammation, oxidative stress, lipotoxicity, and fibrosis, thereby contributing to adverse ventricular remodeling following AMI (11, 12). These features suggest that EAT serves as a critical mediator linking metabolic dysregulation to cardiac structural and functional alterations. Mechanistically, excessive EAT secretes bioactive molecules such as pro-inflammatory cytokines, free fatty acids, and profibrotic factors, which directly act on the adjacent myocardium through paracrine and vasocrine pathways. This interaction promotes myocardial inflammation, oxidative stress, and extracellular matrix remodeling, thereby contributing to ventricular remodeling and adverse cardiovascular events (12). However, most previous studies have primarily focused on EAT volume or thickness, which provide only limited and macroscopic information. Previous population-based studies have reported that the median EAT volume in healthy individuals is approximately 60–70 cm³, with higher values associated with increased cardiovascular risk, highlighting the clinical relevance of EAT burden (13). Such conventional measurements fail to capture the spatial heterogeneity, compositional complexity, and microstructural alterations of adipose tissue, which may be critical determinants of its biological activity. Therefore, a more refined and quantitative characterization of EAT is required to better elucidate its role in post-infarction remodeling and to improve its prognostic value.

Radiomics has emerged as a novel and powerful imaging analysis approach that enables the high-throughput extraction of quantitative features from standard medical images, including descriptors of intensity, texture, shape, and spatial heterogeneity (14–17). By transforming imaging data into mineable high-dimensional features and integrating them with machine learning algorithms, radiomics has shown great promise in enhancing risk stratification and clinical decision-making across a variety of diseases, including cardiovascular disorders (17). Importantly, radiomics provides a non-invasive means to capture subtle tissue-level characteristics that are not discernible through conventional imaging interpretation. However, existing radiomics studies in cardiovascular disease have predominantly focused on myocardial tissue or coronary plaque features, while the potential of EAT-derived radiomics features remains largely unexplored (16). This gap limits a comprehensive understanding of the role of pericardiac adipose tissue in post-infarction remodeling and highlights an unmet need for integrating metabolic and structural imaging information. Given the close biological interaction between EAT and the myocardium, as well as the ability of radiomics to quantify tissue heterogeneity, incorporating EAT-derived radiomics features into predictive models may provide a more precise and biologically relevant approach for early risk assessment after AMI.

In this study, we primarily integrated EAT-derived imaging features with clinical parameters to develop radiomics-based predictive models for VR and MACE. Besides, we conducted *in vivo* (i.e., a high-fat diet (HFD) plus isoproterenol-induced rat model) and *in vitro* (i.e., an EAT-conditioned medium (EAT-CM)-treated H9c2 hypoxia/reoxygenation (H/R) model) experiments to explore whether EAT-derived factors exacerbate myocardial injury and contribute to adverse remodeling under metabolic and ischemic stress. Through the above approach, we finally established a more comprehensive framework for early risk stratification after AMI, which offers predictive and mechanistic insights into post-infarction ventricular remodeling.

## 2 Materials and methods

### 2.1 Study design

This study was designed as a single-center prospective observational cohort study, in which patients diagnosed with acute myocardial infarction (AMI) were enrolled at the Second Affiliated Hospital of Anhui Medical University between January 2022 and June 2023. In addition, *in vivo* and *in vitro* experimental validations were conducted to explore the biological effects of EAT on myocardial injury.

### 2.2 Ethical considerations

All procedures involving human participants and animals were approved by the Institutional Ethics Committee of the Second Affiliated Hospital of Anhui Medical University (YX2022-001). The study adhered to the Declaration of Helsinki and relevant animal welfare guidelines. Clinical trial registration: This study was registered in the Chinese Clinical Trial Registry (ChiCTR2400082074).

### 2.3 Setting and participants

**Figure 1:**
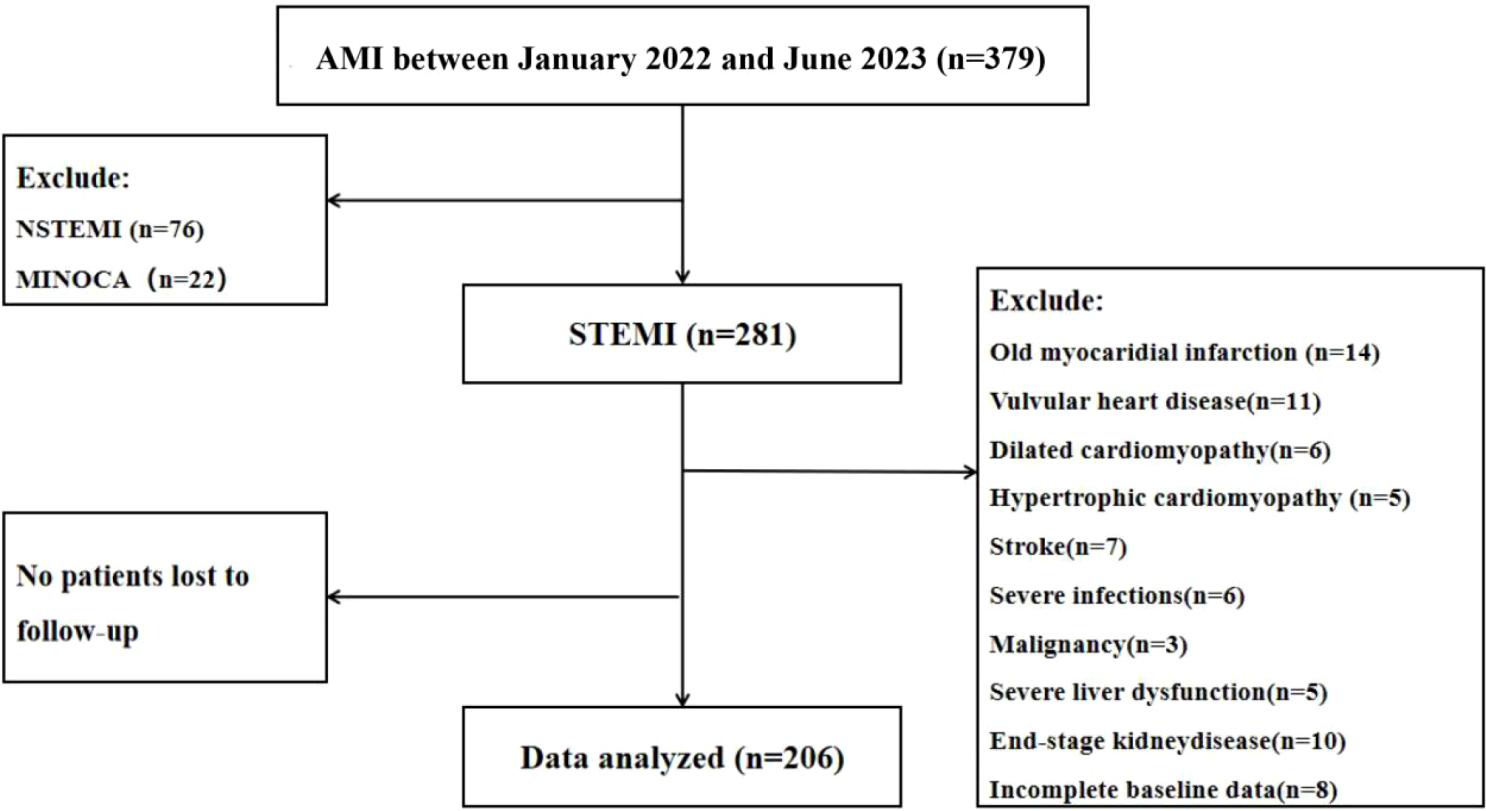
Study flow chart.

Enrolled Patients from the Department of Cardiology at the Second Affiliated Hospital of Anhui Medical University who met the following criteria were included (1): Diagnosis of AMI according to the 4^th^ edition of the Global Definition of Acute Myocardial infarction (18), confirmed by coronary angiography (2); age between 30–75 years (selected based on epidemiological data highlighting AMI incidence in this age group) (3); successful percutaneous coronary intervention within 24 hours of AMI onset; and (4) Door-to-wire (D2W) time < 90 min (defined as the interval from hospital arrival to guidewire crossing the culprit lesion). Patients who met the following criteria were excluded: (1) Non-ST segment elevation myocardial infarction (NSTEMI); (2) myocardial infarction with non-obstructive coronary atherosclerosis (MINOCA); (3) prior history of old myocardial infarction; (4) patients with non-ischemic cardiomyopathy, myocarditis, aortic dissection, rheumatic heart disease, or valvular heart disease; (5) presence of severe cerebrovascular disease, liver dysfunction, infectious diseases, autoimmune diseases, tumors, and advanced chronic kidney disease; and (6) incomplete baseline clinical information or loss to initial follow-up. The flowchart of the study was presented in **Figure 1**.

### 2.4 Sample size determination

206 patients were enrolled, such that the sample size exceeded the minimum requirement estimated based on the events per variable (EPV) principle. The formula for calculating minimum sample size was N ≥ 10 × k/p, where k represents the number of predictor variables and p represents the expected event rate, ensuring that we maintained at least 10 events per predictor in multivariable modeling. Considering previously reported incidences of ventricular remodeling (~30%) and MACE (~25%) post-AMI (19), at least 167 and 160 patients, respectively, were deemed necessary to support the predictive models.

### 2.5 Data collection tools and techniques

#### 2.5.1 Demographic and clinical data collection tool

In order to systematically record patient information, we utilized a structured demographic and clinical data collection form. The form was reviewed by two experienced cardiologists. As all variables were obtained from objective clinical records, reliability was considered inherently reproducible. The recorded variables included age, sex, body mass index, smoking status, alcohol consumption, history of hypertension, diabetes, and coronary artery disease, as well as clinical parameters such as mean arterial pressure, Killip classification, atrial fibrillation, ventricular arrhythmia, atrioventricular block, Global Registry of Acute Coronary Events (GRACE) score, and postoperative medication use. Post-AMI medical treatments were administered according to current clinical guidelines, and relevant treatment information was recorded. All variables were recorded as categorical or continuous variables and were used for subsequent regression and machine learning analyses.

#### 2.5.2 Laboratory assessment

Laboratory parameters included routine hematological and biochemical indices (hemoglobin, platelet count, platelet distribution width, serum creatinine, blood urea nitrogen, alanine transaminase, aspartate transaminase, total bilirubin, lactate dehydrogenase, cystatin C), metabolic and endocrine indicators [glycosylated hemoglobin, uric acid, total cholesterol, triglycerides, low-density lipoprotein (LDL)., high-density lipoprotein (HDL), homocysteine, lipoprotein (a), free triiodothyronine, free thyroxine, thyroid-stimulating hormone], myocardial injury biomarkers [N-terminal B-type natriuretic peptide, high-sensitivity cardiac troponin I (hs-cTnI), myoglobin, creatine kinase], coagulation indicators (D-dimer, fibrin degradation product), and inflammatory markers (neutrophil-to-lymphocyte ratio, C-reactive protein). These laboratory indicators were used to comprehensively assess metabolic status, organ function, systemic inflammation, and myocardial injury.

#### 2.5.3 Echocardiographic assessment

Echocardiographic parameters included left ventricular ejection fraction (LVEF), LV end-diastolic volume (LVEDV), LV end-systolic volume (LVESV), LV diastolic function (LVDF), interventricular septal thickness, LV posterior wall thickness, and LV wall motion dyskinesia index (LVWDI).

#### 2.5.4 Intraoperative and electrocardiographic assessment

Intraoperative imaging indicators included the artery occlusion site, number of diseased branches, presence of slow flow/no-reflow/slow-reflow, hypotension, ventricular arrhythmia during the procedure, and final Thrombolysis in Myocardial Infarction (TIMI) flow classification. These indicators were used to evaluate coronary lesion characteristics, procedural conditions, and myocardial perfusion status. The electrocardiographic DETERMINE score was calculated as the number of leads with [Q waves × 2]+ [fragmented QRS]+[T wave inversion] (20). The assignment of these variables was presented in the **Supplementary Table 1**.

#### 2.5.5 Outcome definition

The primary outcome was VR (**Table 1**), defined as a ≥ 20% increase in LVEDV on echocardiography compared to baseline echocardiography (6). The secondary outcome was MACE (**Table 2**), defined as a composite of heart failure, recurrent angina or myocardial infarction, malignant ventricular arrhythmia, or all-cause mortality.

**Table 1:** Clinical baseline data characteristics of VR outcome.

Variables	Total (n = 206)	Non-VR (n = 145)	VR (n = 61)	Statistic	p
EAT volume, Median (Q1,Q3)	106.5 (90.3, 130.0)	101.3 (89.1, 116.6)	134.9 (106.2, 160.5)	-4.819	< 0.001
LVEF, n (%)				45.922	< 0.001
0	148 (71.8)	124 (85.5)	24 (39.3)		
1	32 (15.5)	13 (9)	19 (31.1)		
2	26 (12.6)	8 (5.5)	18 (29.5)		
LVESV, n (%)				14.552	< 0.001
0	180 (87.4)	135 (93.1)	45 (73.8)		
1	26 (12.6)	10 (6.9)	16 (26.2)		
LVEDV, n (%)				Fisher	0.165
0	196 (95.1)	140 (96.6)	56 (91.8)		
1	10 (4.9)	5 (3.4)	5 (8.2)		
IVS, Median (Q1,Q3)	10.00 (9.00, 11.00)	10.00 (9.00, 11.00)	10.00 (9.00, 12.00)	-1.951	0.051
LVPW, Median (Q1,Q3)	9.00 (9.00, 10.00)	9.00 (9.00, 10.00)	10.00 (9.00, 10.00)	-0.954	0.340
LVWDI, Median (Q1,Q3)	2.00 (0.00, 6.00)	1.00 (0.00, 3.00)	6.00 (4.00, 8.00)	-7.455	< 0.001
Determine score, Median (Q1,Q3)	6.00 (4.00, 10.00)	6.00 (4.00, 8.00)	9.00 (6.00, 12.00)	-4.738	< 0.001
NLR, Median (Q1,Q3)	3.66 (2.29, 7.74)	3.35 (2.24, 6.28)	6.06 (2.79, 9.30)	-2.495	0.013
CRP, Median (Q1,Q3)	2.15 (0.50, 6.57)	2.20 (0.50, 6.80)	2.10 (0.50, 6.20)	-0.457	0.648
HGB, Mean ± SD	142.69 ± 16.38	143.53 ± 15.50	140.70 ± 18.30	1.057	0.293
PLT, Mean ± SD	215.64 ± 67.03	221.26 ± 66.29	202.30 ± 67.43	1.852	0.067
PDW, Median (Q1,Q3)	15.20 (12.00, 16.30)	15.30 (11.80, 16.30)	15.10 (12.80, 16.30)	-0.688	0.492
GOT, Median (Q1,Q3)	32.00 (22.25, 95.75)	31.00 (22.00, 75.00)	43.00 (24.00, 276.00)	-2.195	0.028
GPT, Median (Q1,Q3)	34.00 (25.00, 55.75)	34.00 (25.00, 49.00)	35.00 (23.00, 73.00)	-0.790	0.430
TBIL, Median (Q1,Q3)	12.60 (8.70, 16.98)	12.20 (8.40, 16.50)	13.30 (9.40, 17.00)	-1.000	0.317
LDH, Median (Q1,Q3)	259.00 (194.25, 469.00)	237.00 (182.00, 425.00)	334.00 (236.00, 663.00)	-3.226	0.001
Cr, Median (Q1,Q3)	74.50 (64.00, 88.00)	71.00 (63.00, 87.00)	80.00 (70.00, 90.00)	-1.947	0.051
UA, Median (Q1,Q3)	342.50 (274.25, 395.50)	342.00 (269.00, 394.00)	348.00 (303.00, 404.00)	-0.803	0.422
Cys-C, Median (Q1,Q3)	1.02 (0.77, 1.35)	0.99 (0.74, 1.30)	1.14 (0.89, 1.43)	-1.598	0.110
NT-proBNP, n (%)				Fisher	0.024
0	107 (51.9)	83 (57.2)	24 (39.3)		
1	23 (11.2)	17 (11.7)	6 (9.8)		
2	52 (25.2)	34 (23.4)	18 (29.5)		
3	17 (8.3)	7 (4.8)	10 (16.4)		
4	7 (3.4)	4 (2.8)	3 (4.9)		
Hs-CTnI, n (%)				17.988	< 0.001
0	51 (24.8)	43 (29.7)	8 (13.1)		
1	53 (25.7)	42 (29)	11 (18)		
2	51 (24.8)	35 (24.1)	16 (26.2)		
3	51 (24.8)	25 (17.2)	26 (42.6)		
MYO, n (%)				5.786	0.122
0	67 (32.5)	52 (35.9)	15 (24.6)		
1	40 (19.4)	31 (21.4)	9 (14.8)		
2	53 (25.7)	32 (22.1)	21 (34.4)		
3	46 (22.3)	30 (20.7)	16 (26.2)		
CK-MB, n (%)				11.939	0.008
0	50 (25.7)	44 (30.3)	9 (14.8)		
1	50 (24.3)	39 (26.9)	11 (18)		
2	52 (25.2)	34 (23.4)	18 (29.5)		
3	51 (24.8)	28 (19.3)	23 (37.7)		
D-Dimer, Median (Q1,Q3)	0.29 (0.19, 0.69)	0.25 (0.19, 0.45)	0.39 (0.22, 1.27)	-2.976	0.003
FDP, Median (Q1,Q3)	1.64 (1.13, 2.74)	1.56 (1.07, 2.54)	2.19 (1.27, 4.49)	-2.604	0.009
HB1AC, Median (Q1,Q3)	5.80 (5.50, 6.38)	5.80 (5.50, 6.30)	5.90 (5.50, 6.50)	-0.127	0.899
TC, Median (Q1,Q3)	4.88 (4.18, 5.54)	4.81 (4.15, 5.48)	5.09 (4.26, 5.60)	-1.055	0.292
TG, Median (Q1,Q3)	1.41 (0.95, 2.13)	1.46 (0.95, 2.28)	1.35 (0.98, 1.81)	0.965	0.334
HDL, Median (Q1,Q3)	1.16 (1.00, 1.36)	1.15 (1.00, 1.36)	1.18 (0.97, 1.39)	0.402	0.688
LDL, Median (Q1,Q3)	3.00 (2.32, 3.43)	2.98 (2.33, 3.41)	3.04 (2.30, 3.64)	-0.408	0.683
Lpa, Median (Q1,Q3)	197.00 (101.25, 344.75)	185.00 (101.00, 318.00)	222.00 (103.00, 428.00)	-0.966	0.334
HCY, Median (Q1,Q3)	15.15 (12.50, 19.95)	15.40 (12.50, 19.80)	15.10 (12.90, 20.00)	-0.181	0.857
HR, Median (Q1,Q3)	75.00 (70.00, 87.75)	74.00 (70.00, 86.00)	79.00 (70.00, 96.00)	-1.569	0.117
MAP, Mean ± SD	97.78 ± 14.35	97.81 ± 14.70	97.69 ± 13.62	0.059	0.953
No reflow/slow blood flow, n (%)				17.367	< 0.001
0	152 (73.8)	119 (82.1)	33 (54.1)		
1	54 (26.2)	26 (17.9)	28 (45.9)		
Hypotension, n (%)				0.019	0.890
0	180 (87.4)	127 (87.6)	53 (86.9)		
1	26 (12.6)	18 (12.4)	8 (13.1)		
Ventricular arrhythmia, n (%)				0.012	0.912
0	185 (89.8)	130 (89.7)	55 (90.2)		
1	21 (10.2)	15 (10.3)	6 (9.8)		
TIMI blood flow classification, n (%)				4.912	0.027
0	184 (89.3)	134 (92.4)	50 (82)		
1	22 (10.7)	11 (7.6)	11 (18)		
Artery occlusion site, n (%)				Fisher	0.318
0	119 (57.8)	80 (55.2)	39 (63.9)		
1	85 (41.3)	64 (44.1)	21 (34.4)		
2	2 (1)	1 (0.7)	1 (1.6)		
Number of diseased branches, n (%)				1.210	0.546
0	103 (50)	70 (48.3)	33 (54.1)		
1	71 (34.5)	50 (34.5)	21 (34.4)		
2	32 (15.5)	25 (17.2)	7 (11.5)		
Killip classification, n (%)				Fisher	< 0.001
1	136 (66)	110 (75.9)	26 (42.6)		
2	41 (19.9)	24 (16.6)	17 (27.9)		
3	25 (12.1)	11 (7.6)	14 (23)		
4	4 (1.9)	0 (0)	4 (6.6)		
Atrial fibrillation, n (%)				Fisher	0.007
0	194 (94.2)	141 (97.2)	53 (86.9)		
1	12 (5.8)	4 (2.8)	8 (13.1)		
Ventricular arrhythmia_2, n (%)				Fisher	0.836
0	158 (76.7)	112 (77.2)	46 (75.4)		
1	38 (18.4)	27 (18.6)	11 (18)		
2	6 (2.9)	4 (2.8)	2 (3.3)		
3	4 (1.9)	2 (1.4)	2 (3.3)		
Atrioventricular block, n (%)				Fisher	1.000
0	164 (79.6)	115 (79.3)	49 (80.3)		
1	35 (17)	25 (17.2)	10 (16.4)		
2	7 (3.4)	5 (3.4)	2 (3.3)		
HTN history, n (%)				0.168	0.682
0	80 (38.8)	55 (37.9)	25 (41)		
1	126 (61.2)	90 (62.1)	36 (59)		
DM history, n (%)				1.419	0.234
0	147 (71.4)	107 (73.8)	40 (65.6)		
1	59 (28.6)	38 (26.2)	21 (34.4)		
CAD family history, n (%)				Fisher	0.441
0	198 (96.1)	138 (95.2)	60 (98.4)		
1	8 (3.9)	7 (4.8)	1 (1.6)		
Age, n (%)				2.486	0.288
0	55 (26.7)	43 (29.7)	12 (19.7)		
1	112 (54.4)	77 (53.1)	35 (57.4)		
2	39 (18.9)	25 (17.2)	14 (23)		
Gender, n (%)				0.106	0.745
0	166 (80.6)	116 (80)	50 (82)		
1	40 (19.4)	29 (20)	11 (18)		
BMI, n (%)				Fisher	0.244
0	4 (1.9)	3 (2.1)	1 (1.6)		
1	82 (39.8)	62 (42.8)	20 (32.8)		
2	79 (38.3)	56 (38.6)	23 (37.7)		
3	41 (19.9)	24 (16.6)	17 (27.9)		
DtoW time, Median (Q1,Q3)	75.00 (63.00, 96.00)	74.00 (62.00, 96.00)	78.00 (68.00, 103.00)	-1.086	0.278
Smoking, n (%)				0.174	0.676
0	117 (56.8)	81 (55.9)	36 (59)		
1	89 (43.2)	64 (44.1)	25 (41)		
Drinking, n (%)				0.078	0.780
0	181 (87.9)	128 (88.3)	53 (86.9)		
1	25 (12.1)	17 (11.7)	8 (13.1)		
Onset time, Median (Q1,Q3)	12.00 (8.00, 16.00)	12.00 (8.00, 15.00)	13.00 (8.00, 17.00)	-0.918	0.359

Data are expressed as mean ± standard deviation (SD), median (Q1–Q3), or number (%). Continuous variables were compared using the independent-samples t test or Mann–Whitney U test, as appropriate. Categorical variables were compared using the chi-square test or Fisher’s exact test. A two-sided P < 0.05 was considered statistically significant.

**Table 2:** Baseline clinical characteristics of MACE outcome.

Variables	Total (n = 206)	Non-MACE (n = 165)	MACE (n = 41)	Statistic	p
EAT volume	106.5 (90.3, 130.0)	102.7 (89.1, 122.3)	130.0 (111.8, 162.8)	-3.948	< 0.001
LVEF, n (%)				73.276	< 0.001
0	148 (71.8)	138 (83.6)	10 (24.4)		
1	32 (15.5)	21 (12.7)	11 (26.8)		
2	26 (12.6)	6 (3.6)	20 (48.8)		
LVESV, n (%)				32.357	< 0.001
0	180 (87.4)	155 (93.9)	25 (61)		
1	26 (12.6)	10 (6.1)	16 (39)		
LVEDV, n (%)				Fisher	0.005
0	196 (95.1)	161 (97.6)	35 (85.4)		
1	10 (4.9)	4 (2.4)	6 (14.6)		
IVS	10.00 (9.00, 11.00)	10.00 (9.00, 11.00)	10.00 (9.00, 12.00)	-1.184	0.236
LVPW	9.00 (9.00, 10.00)	9.00 (9.00, 10.00)	9.00 (9.00, 10.00)	-0.217	0.829
LVWDI	2.00 (0.00, 6.00)	1.00 (0.00, 4.00)	7.00 (4.00, 9.00)	-5.792	< 0.001
Determine score	6.00 (4.00, 10.00)	6.00 (4.00, 9.00)	9.00 (6.00, 12.00)	-3.430	< 0.001
NLR	3.66 (2.29, 7.74)	3.53 (2.23, 7.08)	6.06 (2.79, 8.30)	-2.267	0.023
CRP	2.15 (0.50, 6.57)	2.00 (0.50, 6.40)	2.30 (0.50, 8.70)	-0.697	0.486
HGB	142.69 ± 16.38	143.23 ± 15.23	140.54 ± 20.46	0.790	0.433
PLT	215.64 ± 67.03	219.01 ± 66.60	202.07 ± 67.85	1.436	0.156
PDW	15.20 (12.00, 16.30)	15.30 (11.90, 16.30)	14.70 (12.10, 16.30)	0.047	0.963
GOT	32.00 (22.25, 95.75)	31.00 (22.00, 80.00)	45.00 (24.00, 231.00)	-1.517	0.129
GPT	34.00 (25.00, 55.75)	34.00 (25.00, 51.00)	33.00 (24.00, 73.00)	-0.423	0.672
TBIL	12.60 (8.70, 16.98)	12.30 (8.50, 16.70)	13.30 (9.40, 17.10)	-0.865	0.387
LDH	259.00 (194.25, 469.00)	256.00 (187.00, 443.00)	280.00 (211.00, 691.00)	-1.998	0.046
Cr	74.50 (64.00, 88.00)	74.00 (64.00, 88.00)	76.00 (64.00, 88.00)	-0.165	0.869
UA	342.50 (274.25, 395.50)	342.00 (270.00, 397.00)	345.00 (303.00, 380.00)	-0.430	0.667
Cys-C	1.02 (0.77, 1.35)	1.02 (0.78, 1.35)	1.02 (0.69, 1.42)	-0.102	0.918
NT-proBNP, n (%)				Fisher	0.007
0	107 (51.9)	90 (54.5)	17 (41.5)		
1	23 (11.2)	20 (12.1)	3 (7.3)		
2	52 (25.2)	43 (26.1)	9 (22)		
3	17 (8.3)	9 (5.5)	8 (19.5)		
4	7 (3.4)	3 (1.8)	4 (9.8)		
Hs-CTnI, n (%)				3.908	0.272
0	51 (24.8)	43 (26.1)	8 (19.5)		
1	53 (25.7)	44 (26.7)	9 (22)		
2	51 (24.8)	42 (25.5)	9 (22)		
3	51 (24.8)	36 (21.8)	15 (36.6)		
MYO, n (%)				2.513	0.473
0	67 (32.5)	54 (32.7)	13 (31.7)		
1	40 (19.4)	35 (21.2)	5 (12.2)		
2	53 (25.7)	42 (25.5)	11 (26.8)		
3	46 (22.3)	34 (20.6)	12 (29.3)		
CK-MB, n (%)				7.862	0.049
0	53 (25.7)	48 (29.1)	5 (12.2)		
1	50 (24.3)	42 (25.5)	8 (19.5)		
2	52 (25.2)	39 (23.6)	13 (31.7)		
3	51 (24.8)	36 (21.8)	15 (36.6)		
D-Dimer	0.29 (0.19, 0.69)	0.26 (0.19, 0.54)	0.39 (0.20, 1.11)	-2.057	0.040
FDP	1.64 (1.13, 2.74)	1.58 (1.13, 2.74)	1.95 (1.17, 4.10)	-1.237	0.216
HB1AC	5.80 (5.50, 6.38)	5.80 (5.50, 6.40)	5.80 (5.40, 6.30)	0.540	0.589
TC	4.88 (4.18, 5.54)	4.94 (4.17, 5.53)	4.82 (4.22, 5.58)	0.110	0.913
TG	1.41 (0.95, 2.13)	1.48 (0.97, 2.20)	1.14 (0.91, 1.49)	2.057	0.040
HDL	1.16 (1.00, 1.36)	1.15 (1.00, 1.36)	1.20 (0.99, 1.36)	-0.034	0.973
LDL	3.00 (2.32, 3.43)	2.99 (2.39, 3.42)	3.01 (2.20, 3.64)	-0.007	0.994
Lpa	197.00 (101.25, 344.75)	204.00 (102.00, 338.00)	167.00 (93.00, 429.00)	0.020	0.984
HCY	15.15 (12.50, 19.95)	15.20 (12.40, 19.80)	15.10 (13.20, 20.00)	-0.445	0.656
HR	75.00 (70.00, 87.75)	75.00 (70.00, 86.00)	75.00 (69.00, 98.00)	-0.984	0.325
MAP	97.78 ± 14.35	98.65 ± 14.81	94.27 ± 11.88	2.005	0.049
No reflow/slow blood flow, n (%)				6.155	0.013
0	152 (73.8)	128 (77.6)	24 (58.5)		
1	54 (26.2)	37 (22.4)	17 (41.5)		
Hypotension, n (%)				0.381	0.537
0	180 (87.4)	143 (86.7)	37 (90.2)		
1	26 (12.6)	22 (13.3)	4 (9.8)		
Ventricular arrhythmia, n (%)				Fisher	0.773
0	185 (89.8)	147 (89.1)	38 (92.7)		
1	21 (10.2)	18 (10.9)	3 (7.3)		
TIMI blood flow classification, n (%)				Fisher	0.004
0	184 (89.3)	153 92.7)	31 75.6)		
1	22 (10.7)	12 (7.3)	10 (24.4)		
Artery occlusion site, n (%)				Fisher	0.133
0	119 (57.8)	90 (54.5)	29 (70.7)		
1	85 (41.3)	73 (44.2)	12 (29.3)		
2	2 (1)	2 (1.2)	0 (0)		
Number of diseased branches, n (%)				1.842	0.398
0	103 (50)	83 (50.3)	20 (48.8)		
1	71 (34.5)	54 (32.7)	17 (41.5)		
2	32 (15.5)	28 (17)	4 (9.8)		
Killip classification, n (%)				Fisher	0.013
1	136 (66)	115 (69.7)	21 (51.2)		
2	41 (19.9)	31 (18.8)	10 (24.4)		
3	25 (12.1)	18 (10.9)	7 (17.1)		
4	4 (1.9)	1 (0.6)	3 (7.3)		
Atrial fibrillation, n (%)				Fisher	0.016
0	194 (94.2)	159 (96.4)	35 (85.4)		
1	12 (5.8)	6 (3.6)	6 (14.6)		
Ventricular arrhythmia_2, n (%)				Fisher	0.737
0	158 (76.7)	127 (77)	31 (75.6)		
1	38 (18.4)	31 (18.8)	7 (17.1)		
2	6 (2.9)	4 (2.4)	2 (4.9)		
3	4 (1.9)	3 (1.8)	1 (2.4)		
Atrioventricular block, n (%)				Fisher	0.770
0	164 (79.6)	131 (79.4)	33 (80.5)		
1	35 (17)	29 (17.6)	6 (14.6)		
2	7 (3.4)	5 (3)	2 (4.9)		
HTN history, n (%)				0.553	0.457
0	80 (38.8)	62 (37.6)	18 (43.9)		
1	126 (61.2)	103 (62.4)	23 (56.1)		
DM history, n (%)				1.581	0.209
0	147 (71.4)	121 (73.3)	26 (63.4)		
1	59 (28.6)	44 (26.7)	15 (36.6)		
CAD family history, n (%)				Fisher	0.361
0	198 (96.1)	157 (95.2)	41 (100)		
1	8 (3.9)	8 (4.8)	0 (0)		
Age, n (%)				7.085	0.029
0	55 (26.7)	50 (30.3)	5 (12.2)		
1	112 (54.4)	88 (53.3)	24 (58.5)		
2	39 (18.9)	27 (16.4)	12 (29.3)		
Gender, n (%)				0.000	0.986
0	166 (80.6)	133 (80.6)	33 (80.5)		
1	40 (19.4)	32 (19.4)	8 (19.5)		
BMI, n (%)				Fisher	0.956
0	4 (1.9)	3 (1.8)	1 (2.4)		
1	82 (39.8)	66 (40)	16 (39)		
2	79 (38.3)	64 (38.8)	15 (36.6)		
3	41 (19.9)	32 (19.4)	9 (22)		
DtoW time	75.00 (63.00, 96.00)	74.00 (63.00, 96.00)	80.00 (69.00, 96.00)	-0.852	0.394
Smoking, n (%)				0.063	0.802
0	117 (56.8)	93 (56.4)	24 (58.5)		
1	89 (43.2)	72 (43.6)	17 (41.5)		
Drinking, n (%)				Fisher	1.000
0	181 (87.9)	145 (87.9)	36 (87.8)		
1	25 (12.1)	20 (12.1)	5 (12.2)		
Onset time	12.00 (8.00, 16.00)	12.00 (8.00, 15.00)	13.00 (9.00, 17.00)	-0.856	0.392

Data are expressed as mean ± standard deviation (SD), median (Q1–Q3), or number (%). Continuous variables were compared using the independent-samples t test or Mann–Whitney U test, as appropriate. Categorical variables were compared using the chi-square test or Fisher’s exact test. A two-sided P < 0.05 was considered statistically significant.

Patients were followed monthly via standardized telephone interviews and outpatient visits for a period of one year. Events were independently reviewed and adjudicated by clinicians blinded to imaging data and EAT measurements. Participants lost during the initial follow-up were excluded from the final analysis.

### 2.6 Cardiac CT acquisition and EAT segmentation

#### 2.6.1 Cardiac CT acquisition and EAT segmentation

CCTA was performed using Philips Brilliance iCT and IQon Spectral CT (Philips Healthcare), covering the heart region from 1 cm below the tracheal bifurcation to the cardiac base. The patients were instructed to hold their breath for 6 s before initiating the scan, with the iCT and IQon tube voltages set at 100/120 kV and 120 kV, respectively. The rotation time was 0.27 s, and the collimation was set to 128 mm × 0.625 mm for iCT and 64 mm × 0.625 mm for IQon Spectral CT. The field of view was 250 mm × 250 mm, and the reconstruction thickness and slice interval were 0.9 mm and 0.45 mm, respectively.

EAT segmentation was performed using 3D Slicer (v5.2.0) following established protocols (21). A semi-automated approach was applied to delineate EAT within the pericardial contour using a Hounsfield Unit (HU) threshold of −200 to +30 HU. Boolean operations were used to isolate adipose tissue, and anatomical landmarks (e.g., pleural reflections, left atrial wall) were referenced in cases of pericardial boundary ambiguity. Manual corrections were performed when necessary to exclude myocardium, vessels, or artifacts.

Segmentation was independently conducted by two experienced radiologists blinded to clinical outcomes. Inter-observer agreement was assessed using the intraclass correlation coefficient (ICC), demonstrating excellent reliability. A representative example of EAT segmentation is shown in **Supplementary Figure 1**.

#### 2.6.2 Radiomics feature extraction and Rad-score construction

Radiomics features were extracted from the segmented EAT regions on CCTA images using Pyradiomics (https://pyradiomics.readthedocs.io/en/latest/) according to the guidelines of the Imaging Biomarker Standardization Initiative (IBSI) (15). Before feature extraction, images were preprocessed by resampling to a uniform voxel size (1×1×1 mm³) and normalizing intensity to a standard grayscale. A total of seven categories of radiomics features were computed from original and transformed images, including first-order statistical features of the original image, shape-based features, and texture-based features derived from gray-level co-occurrence matrices (GLCM), gray-level run-length matrices (GLRLM), gray-level size-zone matrices (GLSZM), gray-level dependence matrices (GLDM), and neighboring gray tone difference matrices (NGTDM).

All radiomics feature variable was standardized using z-scores normalization, calculated as z = (x−x_mean_)/x_sd_, to ensure comparability. Feature selection was performed via the Least Absolute Shrinkage and Selection Operator (LASSO) regression, with the optimal regularization parameter (λ) determined by 10-fold cross-validation to minimize prediction error (22).

Considering the limited sample size, stratified sampling based on clinical outcomes (VR and MACE occurrence) was employed to maintain outcome balance within training and validation sets. Three widely-used machine learning algorithms, including Random Forest (RF), k-Nearest Neighbor (KNN), and Night Gradient Boosting Machine (NightGBM), were selected due to their established predictive performance and robustness in medical imaging research.

The Rad-score construction and model validation involved the following steps: 1) Randomly the dataset (n=206) was randomly partitioned into 10 equal parts using stratified sampling; 2) 10-fold cross-validation was conducted, iteratively selecting nine folds as the training dataset and one fold as the validation dataset. Hyperparameters of each algorithm were tuned on training datasets based on the Area Under the Receiver Operating Characteristic (ROC) curve (AUC); 3) optimal hyperparameters were used to generate predictive probabilities for the validation fold; 4) After completing 10 iterations, predictive probabilities from all validation folds were pooled to calculate the overall model performance (AUC). 5) Based on the highest AUC, the optimal algorithm was selected, and the Rad-score was computed as a weighted sum of the selected radiomics features, with feature weights determined by their predictive importance within the optimal algorithm.

### 2.7 Machine learning model construction and evaluation

Two predictive models were developed and assessed in the study. The Model (1) included clinical parameters and EAT volume, while Model (2) combined clinical parameters, EAT volume, and the derived Rad-score. Model discrimination capability was evaluated using ROC curves and time-dependent C-index. Calibration curves assessed the agreement between predicted probabilities and observed outcomes. Decision Curve Analysis (DCA) evaluated the net clinical benefits of each model at different threshold probabilities, highlighting their clinical applicability. Internal validation of the models was performed using bootstrap resampling (1,000 iterations), a well-established approach to estimate model optimism and prevent overfitting.

### 2.8 *In vivo* experimental validation

#### 2.8.1 Animal model and group assignment

Eight-week-old male Sprague-Dawley (SD) rats (220–250 g, SPF grade) were housed under controlled environmental conditions (22 ± 2 °C, 55 ± 5% humidity, 12-h light/dark cycle) with free access to standard food and water. All animal experiments were approved by the Institutional Animal Care and Use Committee of Guangdong Medical Laboratory Animal Center(D202508-11) and conducted in accordance with the National Institutes of Health Guide for the Care and Use of Laboratory Animals and the ARRIVE guidelines.

Animals were randomly divided into four experimental groups (n = 9 per group) based on diet and isoproterenol (ISO) administration (1): ND: received a standard chow diet for 8 weeks; (2) ND+ISO group: standard diet plus ISO (85 mg/kg/day, subcutaneous; Cat#I6504, Sigma-Aldrich) for two consecutive days; (3) HFD: high-fat diet (45% kcal from fat; Cat#D12451, Research Diets Inc.) for 8 weeks; (4) HFD+ISO: high-fat diet for 8 weeks plus ISO administration as above. This model was designed to examine the combined impact of metabolic overload and sympathetic stimulation on EAT phenotype and myocardial injury, as previously described (23).

At the end of the intervention, EAT was harvested under aseptic conditions and cultured in serum-free Dulbecco’s Modified Eagle Medium (DMEM, Cat#11965092, Gibco) for 24 hours at 37 °C with 5% CO_2_ in a humidified incubator (Thermo Fisher Scientific, Model: Heracell VIOS 160i). The conditioned medium (EAT-CM) was collected, centrifuged at 3,000 rpm for 10 minutes, and filtered through a 0.22 µm membrane (Cat#SLGP033RS, Millipore) to remove tissue debris, yielding a paracrine-activity-enriched medium for subsequent cellular experiments.

#### 2.8.2 Echocardiography and tissue assessment

Cardiac function was assessed by transthoracic echocardiography (Vevo 3100, FUJIFILM VisualSonics, Toronto, Canada) equipped with a 13–24 MHz linear transducer (MX550D). Rats were anesthetized with 2% isoflurane and placed supine on a temperature-controlled platform. M-mode images were obtained from parasternal long- and short-axis views to measure left ventricular end-diastolic diameter (LVEDD), end-systolic diameter (LVESD), and heart rate (HR). Ejection fraction (EF) and fractional shortening (FS) were automatically calculated using standard formulas. All measurements were averaged over three cardiac cycles and analyzed by an investigator blinded to group allocation.

Following echocardiographic assessment, rats were euthanized under deep anesthesia, and the hearts were rapidly excised, rinsed in cold phosphate-buffered saline (PBS), and blotted dry. EAT surrounding the ventricles was carefully dissected and weighed using an electronic analytical balance. Heart weight (HW) and body weight (BW) were recorded immediately before sacrifice, and the heart weight-to-body weight ratio (HW/BW, mg/g) was calculated as an index of cardiac hypertrophy. All measurements were performed in triplicate by two independent investigators to ensure consistency.

#### 2.8.3 Histopathological analysis

Paraffin-embedded heart tissues were sectioned at 5 µm and processed for standard histological staining. Sections were deparaffinized, rehydrated through graded alcohol, and stained according to the manufacturers’ protocols. All image acquisition and quantitative analyses were performed by two independent investigators blinded to group allocation.

Hematoxylin and eosin (HE) staining was used to assess general myocardial morphology, including cardiomyocyte arrangement, inflammatory cell infiltration, and tissue integrity. After fixation in 4% paraformaldehyde for 24 h, sections were stained with an HE kit (Cat#G1120, Servicebio) and examined under a light microscope (Olympus BX53, Tokyo, Japan). Five representative fields per section were captured for qualitative evaluation.

Wheat germ agglutinin (WGA) staining was applied to determine cardiomyocyte cross-sectional area. Following antigen retrieval, sections were incubated with Alexa Fluor 488-conjugated WGA (Cat#W11261, 1:200 dilution) for 30 min at room temperature in the dark, followed by nuclear counterstaining with DAPI (Cat#G1012, Servicebio). Fluorescence images were acquired using a confocal microscope (Nikon Eclipse C1, Nikon, Japan). Cell borders were outlined, and cross-sectional areas were quantified in five randomly selected fields per section using ImageJ software (NIH, USA).

Masson’s trichrome staining was performed by using a commercial kit (Cat#G1340, Solarbio) to evaluate myocardial fibrosis. Collagen fibers were stained blue, and myocardial tissue red. Fibrotic area was calculated as the ratio of collagen-stained area to the total myocardial area in five random microscopic fields per section using ImageJ software.

#### 2.8.4 Serum biomarker measurement

Serum levels of lactate dehydrogenase (LDH), cardiac troponin I (cTnI), and creatine kinase-MB (CK-MB) were measured to evaluate myocardial injury. Blood samples were collected from the abdominal aorta at the time of sacrifice, left to clot for 30 minutes, and centrifuged at 3,000 rpm for 10 min at 4 °C. Serum was aliquoted and stored at -80 °C until analysis. Biomarker levels were quantified using ELISA kits for LDH (MM-0595R), cTnI (MM-0684R), and CK-MB (MM-0596R) from Jiangsu Meimian Industrial Co., Ltd. (Yancheng, China), following the manufacturer’s instructions. Absorbance was measured using a microplate reader (BioTek Synergy HTX, USA), and concentrations were calculated based on standard curves. All assays were performed in duplicate.

### 2.9 *In vitro* experimental validation

#### 2.9.1 Cell culture and treatment

H9c2 rat cardiomyoblasts (ATCC, CRL-1446) were cultured in complete DMEM supplemented with 10% fetal bovine serum (Cat#10099141C, Gibco) and 1% penicillin-streptomycin (Cat#15140122, Gibco), and maintained at 37 °C in a humidified incubator with 5% CO_2_.

Cells were randomly assigned to four experimental groups: (1) Control group: cells were maintained under normoxic conditions; (2) hypoxia/reoxygenation (H/R) group: cells were subjected to hypoxia (1% O_2_, 5% CO_2_, 94% N_2_) for 6 h in a tri-gas incubator (Binder CB 160), followed by 24 h reoxygenation; (3) EAT-CM group: cells were incubated with EAT-CM for 24 h under normoxia to assess the direct paracrine effect; (4) EAT-CM+H/R group: cells were pretreated with EAT-CM for 24 h, then exposed to the H/R protocol to evaluate combined effects of EAT-derived factors and ischemia/reperfusion injury.

#### 2.9.2 TUNEL assay

Apoptosis of H9c2 cells was assessed using a TUNEL assay kit (Cat#C1086, Beyotime). After treatment, cells on coverslips were fixed with 4% paraformaldehyde for 30 minutes, permeabilized with 0.1% Triton X-100 for 5 min, and incubated with TUNEL working solution according to the manufacturer’s protocol. Nuclei were counterstained with DAPI (Cat#G1012, Servicebio), and images were captured using a confocal microscope (Nikon Eclipse C1, Japan). The apoptotic index was calculated as the percentage of TUNEL-positive nuclei over total nuclei in five randomly selected fields using ImageJ software.

#### 2.9.3 Quantitative real-time PCR

Total RNA was extracted from H9c2 cells using TRIzol reagent (Cat#15596018, Invitrogen) according to the manufacturer’s protocol. RNA concentration and purity were determined using a NanoDrop 2000 spectrophotometer (Thermo Fisher Scientific, USA). Reverse transcription was performed with a cDNA synthesis kit (Cat#R223-01, Vazyme). qRT-PCR was carried out using SYBR Green Master Mix (Cat#Q711-02, Vazyme) on a StepOnePlus Real-Time PCR System (Applied Biosystems, USA). The expression levels of ANP, BNP, and β-MHC were quantified, with GAPDH serving as the internal reference. Relative mRNA expression was calculated using the 2^−ΔΔCt^ method. Primer sequences used in this study are listed in **Supplementary Table 4**.

### 2.10 Statistical analysis

R software (version 3.6.1, MA, USA), Python (version 3.9.12), and SPSS software (version 26.0, IBM Corp., Armonk, NY, USA) were used to perform all statistical analyses. A two-sided P-value < 0.05 was considered statistically significant.

After testing using the Shapiro–Wilk test, continuous variables were presented as mean ± standard deviation (SD) or median (Q1–Q3), as appropriate. For assessing differences between groups, the independent-samples t test was used for normally distributed variables; otherwise, the Mann–Whitney U test was used. Categorical variables were presented as counts (percentages) and compared using the Chi-square or Fisher’s exact test. For multiple-group comparisons in animal and cell experiments, one-way ANOVA (with Bonferroni *post hoc* test) or Kruskal–Wallis test (with Dunn’s multiple comparison) was applied. Kaplan–Meier analysis with log-rank test was used to compare event-free survival.

Least absolute shrinkage and selection operator (LASSO) regression was utilized for feature selection. Independent predictors of VR and major adverse cardiovascular events (MACE) were identified by multivariate Cox proportional hazards models. Concordance index (C-index) and area under the receiver operating characteristic curve (AUC) from cross-validation were applied to evaluate model discrimination. Calibration was assessed with calibration plots, and clinical utility was examined using decision curve analysis (DCA). Shapley additive explanations (SHAP) were employed to explore model interpretability. Variables with <5% missing data were imputed using median (continuous) or mode (categorical) values; variables with >10% missingness were excluded from multivariate analyses.

## 3 Results

### 3.1 Patient characteristics

At last, 206 patients were enrolled in this study, which comprised 166 males and 40 females, whose mean age was 58.2 ± 12.9 years. None of them were lost to follow-up. During the 12-month follow-up, there were 61 patients (29.6%) who developed VR, and 41 patients (19.9%) who experienced major adverse cardiovascular events (MACE). What these events were mainly related to were several key clinical and imaging risk factors, including increased EAT volume, decreased LVEF, higher DETERMINE score, and the presence of no-reflow/slow blood flow, all of which have been implicated in promoting myocardial injury, impaired perfusion, and subsequent ventricular remodeling and adverse cardiovascular outcomes.

Baseline clinical characteristics stratified by VR outcome are presented in **Table 1**. Compared with the non-VR group, patients with VR showed significantly higher EAT volume, LVWDI, DETERMINE score, NLR, GOT, LDH, D-dimer, and FDP levels, as well as higher proportions of elevated hs-cTnI and CK-MB levels, no-reflow/slow blood flow, abnormal TIMI flow classification, advanced Killip classification, and atrial fibrillation (all P < 0.05). Significant differences were also observed in LVEF, LVESV, and NT-proBNP distributions between the two groups (all P < 0.05).

Baseline characteristics stratified by MACE outcome are shown in **Table 2**. Compared with the non-MACE group, patients with MACE had significantly higher EAT volume, LVWDI, DETERMINE score, NLR, LDH, D-dimer, triglyceride levels, and mean arterial pressure, along with higher proportions of CK-MB elevation, no-reflow/slow blood flow, abnormal TIMI flow classification, advanced Killip classification, and atrial fibrillation (all P < 0.05). In addition, significant differences were observed in LVEF, LVESV, LVEDV, NT-proBNP, and age distribution between groups (all P < 0.05).

### 3.2 Construction of predictive model with clinical parameters

Patients were stratified into high- and low-EAT volume groups based on the median EAT volume (106.5 cm^3^) as the threshold value. Notably, the median EAT volume in this cohort was markedly higher than the reported values in healthy populations, further supporting the pathological relevance of increased EAT burden in patients with AMI. Kaplan-Meier survival curve (**Supplementary Figures 2A, B**) showed that patients in the high-EAT volume group had a significantly higher cumulative incidence of VR and MACE compared with those in the low-EAT volume group. During the one-year follow-up, the time-dependent C-index (**Supplementary Figures 2C, D**) confirmed that EAT volume had superior predictive value for VR and MACE compared to BMI (both P<0.05), LDL cholesterol (both P<0.001), hs-CTnI (both P<0.05), and NT-proBNP (both P<0.05), indicating its stronger prognostic utility in risk stratification.

**Figure 2:**
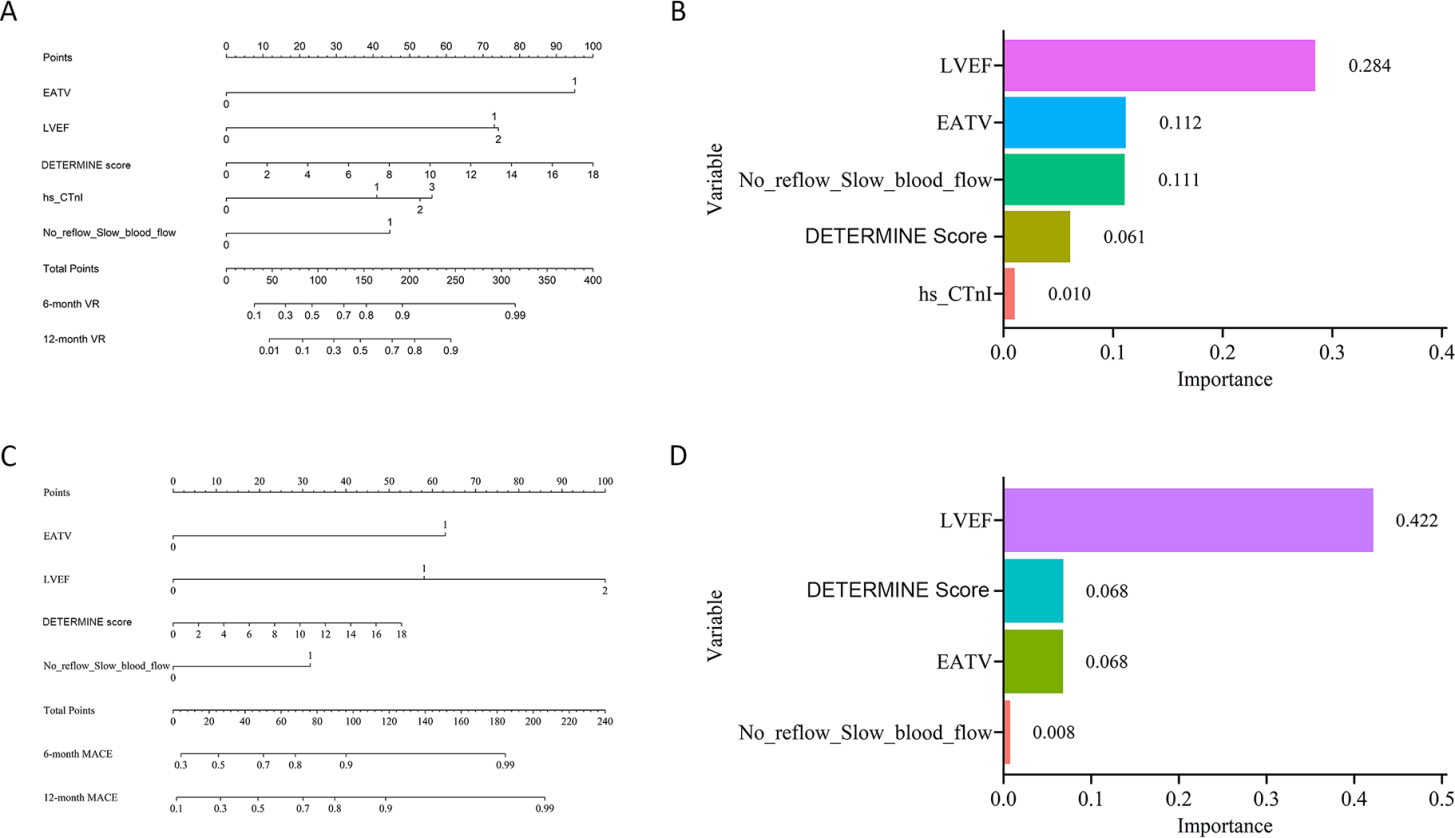
Predictive nomograms and variable importance for ventricular remodeling (VR) and major adverse cardiovascular events (MACE). **(A)** nomogram for predicting 6- and 12-month VR based on clinical and imaging predictors; **(B)** relative importance of each predictor in the VR model; **(C)** nomogram for predicting 6- and 12-month MACE based on clinical and imaging predictors; **(D)** relative importance of each predictor in the MACE model.

LASSO regression for VR identified 10 variables, including EAT volume, LVEF, DETERMINE Score, hs-CTnI, No Reflow/Slow Blood Flow, platelet, lactate dehydrogenase, LVWDI, Killip classification, and body mass index. Among these, the first five variables were confirmed as independent risk factors for VR (**Supplementary Table 2**; **Supplementary Figures 3A, B** ). Similarly, LASSO regression for MACE yielded four non-zero variables, including EAT volume, LVEF, DETERMINE Score, and No Reflow/Slow Blood Flow, all of which were corroborated by the multivariable Cox regression model to be independent risk factors for MACE (**Supplementary Table 3**; **Supplementary Figures 3C, D**). The constructed nomograms (Model 1-VR & Model 1-MACE) and the corresponding contributions from EAT volume and other significant variables for VR and MACE are presented in **Figure 2**, demonstrating the integrated predictive capacity of the selected variables. Kaplan-Meier survival analysis (**Supplementary Figure 4**) showed a significantly higher occurrence of VR and MACE at optimal cut-off points of 213.8 and 128.0, respectively (both P < 0.0001), suggesting that Model 1-VR and Model 1-MACE exhibit favorable discriminative power. The fully adjusted restricted cubic spline analysis showed a nonlinear relationship between the EAT volume and VR or MACE with an inflection point at 106.5 cm^3^ (P for nonlinearity < 0.05) (**Supplementary Figure 5**).

### 3.3 EAT feature selection and explainable Rad-score

**Figure 3:**
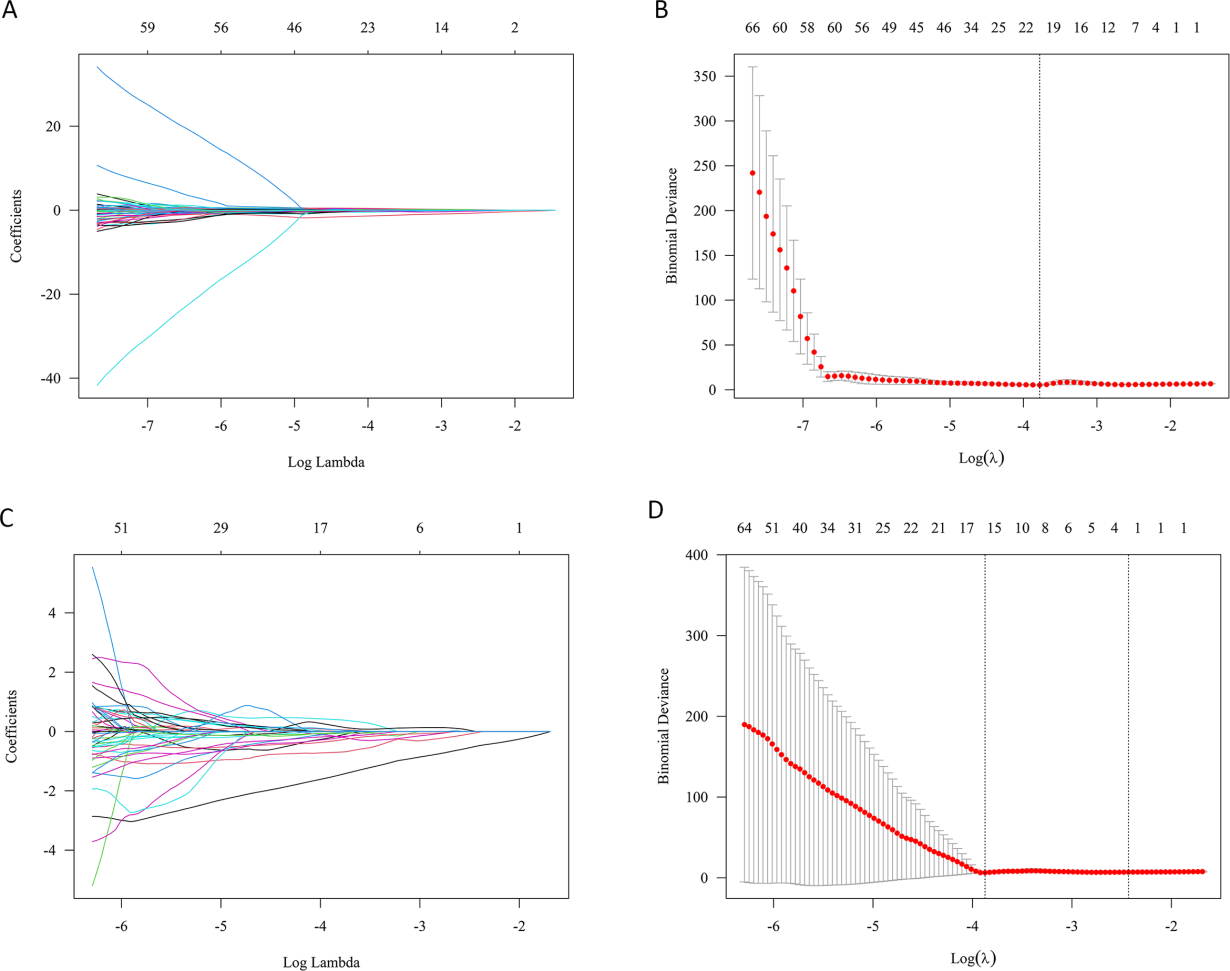
Selection of the epicardial adipose tissue radiomic features using LASSO regression. **(A, C)**: LASSO coefficient profiles for VR **(A)** and MACE **(C)**. As the penalty parameter (λ) increases, more coefficients shrink to zero, leaving only the most predictive features; **(B, D)**: Optimal λ selection using 10-fold cross-validation for VR **(B)** and MACE **(D)**. The vertical dotted line represents the λ value with the minimum binomial deviance, corresponding to the optimal number of non-zero coefficients selected for the predictive model.

A total of 1316 radiomics features were initially extracted. LASSO regression identified analysis identified optimal subsets of radiomics features by selecting a λ value that minimized binomial deviance through 10-fold cross-validation (**Figure 3**). Specifically, 19 features were selected for predicting VR (λ=0.019), and 15 features for predicting MACE (λ=0.024), based on the optimal λ indicated by the dashed vertical lines in **Figures 3B, D**.

Among three evaluated machine learning algorithms, RF achieved the highest performance for predicting VR (AUC = 0.828, 95% CI: 0.765–0.891), while LightGBM performed best for predicting MACE (AUC = 0.790, 95% CI: 0.711–0.859), outperforming the other algorithms (**Supplementary Figure 6**). Consequently, the RF was selected to construct the Rad-score for VR prediction, whereas LightGBM was used for MACE prediction.

**Figure 4:**
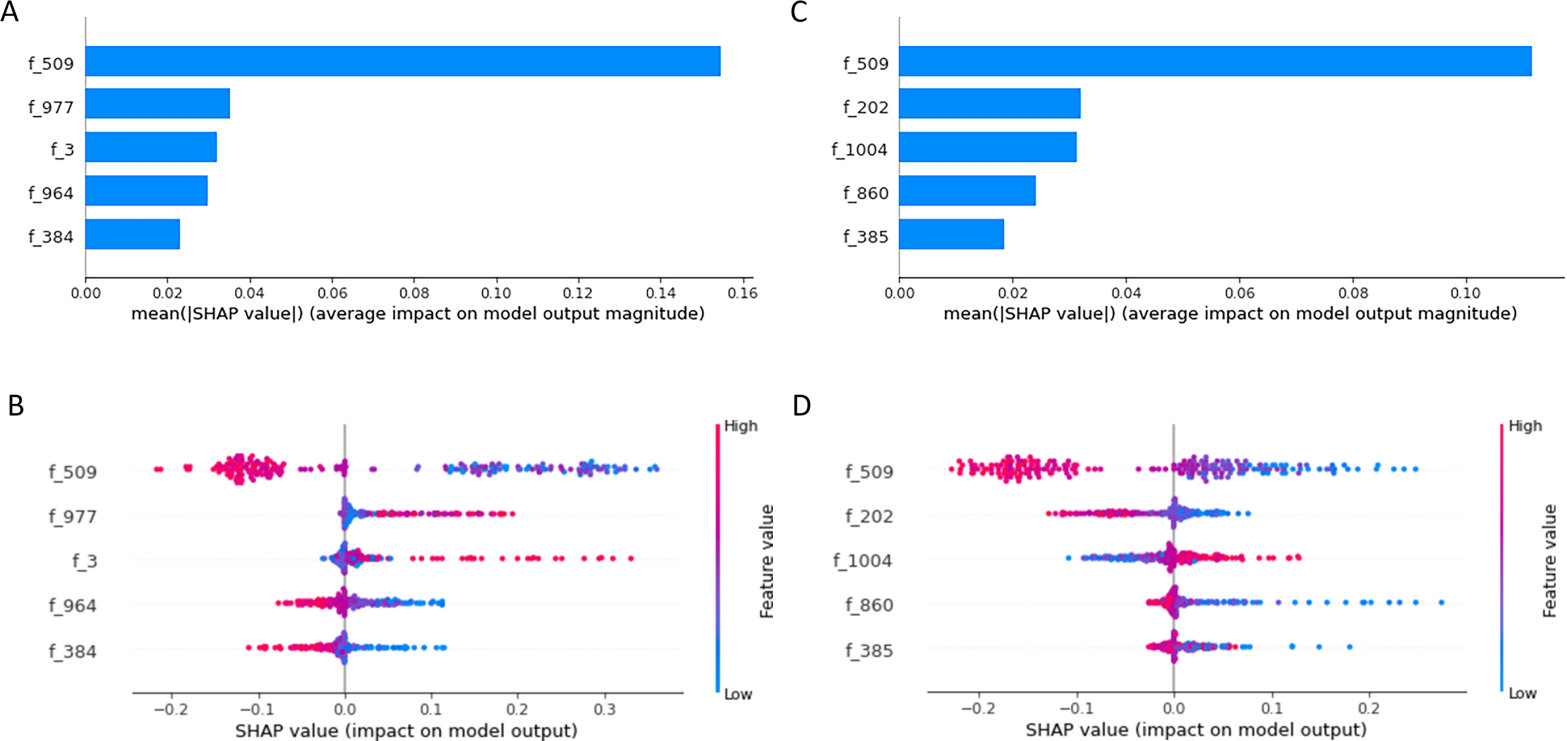
Shapley additive explanations (SHAP) analysis of the top 5 radiomics feature variables for ventricular remodeling (VR) and major adverse cardiovascular events (MACE). **(A, C)**: SHAP bar plots displaying the mean absolute SHAP values, indicating the average impact of each radiomics feature on the predictive model for VR **(A)** and MACE **(C)**. **(B, D)**: SHAP beeswarm plots demonstrating the distribution of SHAP values for individual feature contributions in VR **(B)** and MACE **(D)**.

The SHAP analysis identified the top five radiomic features contributing significantly to the prediction of VR and MACE (**Figure 4**). For predicting VR outcome, the top-ranked features were as follows: f-509 (Log-sigma 5.0mm-3D-GLCM-IDMN), f-977 [Wavelet-HLH-GLCM-Informational Measure of Correlation 2 (IMC2)], f-3 [Original-Shape-Least Axis Length (LAL)], f-964 (Wavelet-HLH-GLCM-Cluster Prominence (24)), and f-384 (Log-sigma 3.0mm-3D-NGTDM-Complexity 2). Similarly, for MACE outcome, the most important features were f-509, f-202 (Log-sigma 2.0mm-3D-First Order-90th Percentile), f-1004 [Wavelet-HLH-GLRLM-High Gray Level Run Emphasis (HGLRE)], f-860 (Wavelet-HLL-First Order-Mean), and f-385 (Log-sigma 3.0mm-3D-NGTDM-Contrast). Notably, feature f-509, representing the texture homogeneity of EAT, demonstrated the highest predictive contribution for both outcomes, suggesting its robust predictive value in assessing cardiovascular risk post-AMI.

### 3.4 Construction of joint predictive models

**Figure 5:**
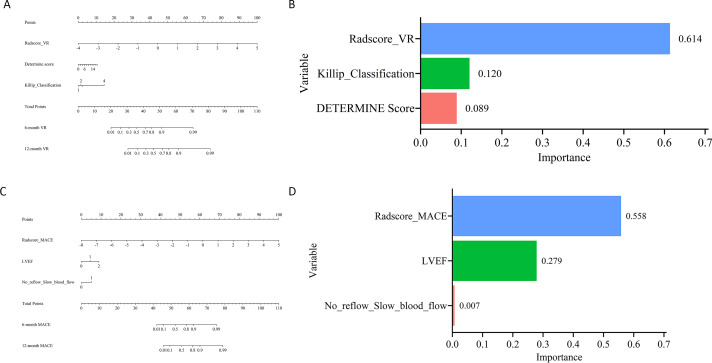
Nomograms and variable importance for predicting ventricular remodeling (VR) and major adverse cardiovascular events (MACE) using radiomics-based models. **(A)** nomogram for predicting VR based on EAT radiomics features combined with clinical parameters; **(B)** relative contribution of each predictor in the VR model; **(C)** nomogram for predicting MACE based on EAT radiomics features combined with clinical parameters; **(D)** relative contribution of each predictor in the MACE model.

Based upon LASSO regression and subsequent Cox multivariate analyses, EAT Rad-score, DETERMINE score, and Killip classification were identified as independent predictors of VR (**Figures 5A, B**). After that, we constructed a nomogram which integrates these variables (Model 2-VR) for predicting VR risk at 6 and 12 months follow-up, and its potential clinical applicability indeed was demonstrated. Likewise, the EAT Rad-score, LVEF, as well as No Reflow/Slow Blood Flow were identified as independent risk factors for the MACE outcome, and they were incorporated into a predictive nomogram (Model 2-MACE, **Figures 5C, D**), which further supports the robustness of the integrated model.

### 3.5 Model validation and comparison

**Figure 6:**
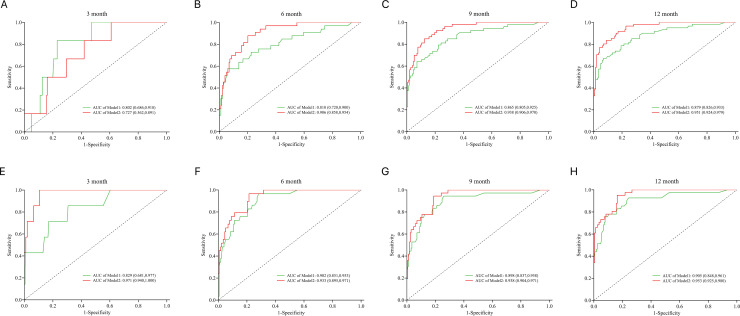
ROC curves comparing the predictive performance of model 1 and model 2 for ventricular remodeling (VR) and major adverse cardiovascular events (MACE) at different follow-up periods. **(A–D)** ROC curves for VR prediction at 3 months **(A)**, 6 months **(B)**, 9 months **(C)**, and 12 months **(D)**; **(E–H)** ROC curves for MACE prediction at 3 months **(E)**, 6 months **(F)**, 9 months **(G)**, and 12 months **(H)**.

Model 2 (integrating Rad-score) revealed superior predictive performance relative to Model 1 (clinical parameters and EAT volume only). As depicted in **Figure 6**, the ROC curve analyses indicated that Model 2-VR had consistently higher AUC values relative to Model 1-VR at 6 months (0.906 vs 0.810), 9 months (0.938 vs 0.865), and 12 months (0.951 vs 0.879) of follow-up, and that Model 2-MACE similarly showed higher AUC values than Model 1-MACE at 3 months (0.971 vs 0.829), 6 months (0.933 vs. 0.902), 9 months (0.938 vs. 0.898), and 12 months (0.953 vs. 0.905).

**Figure 7:**
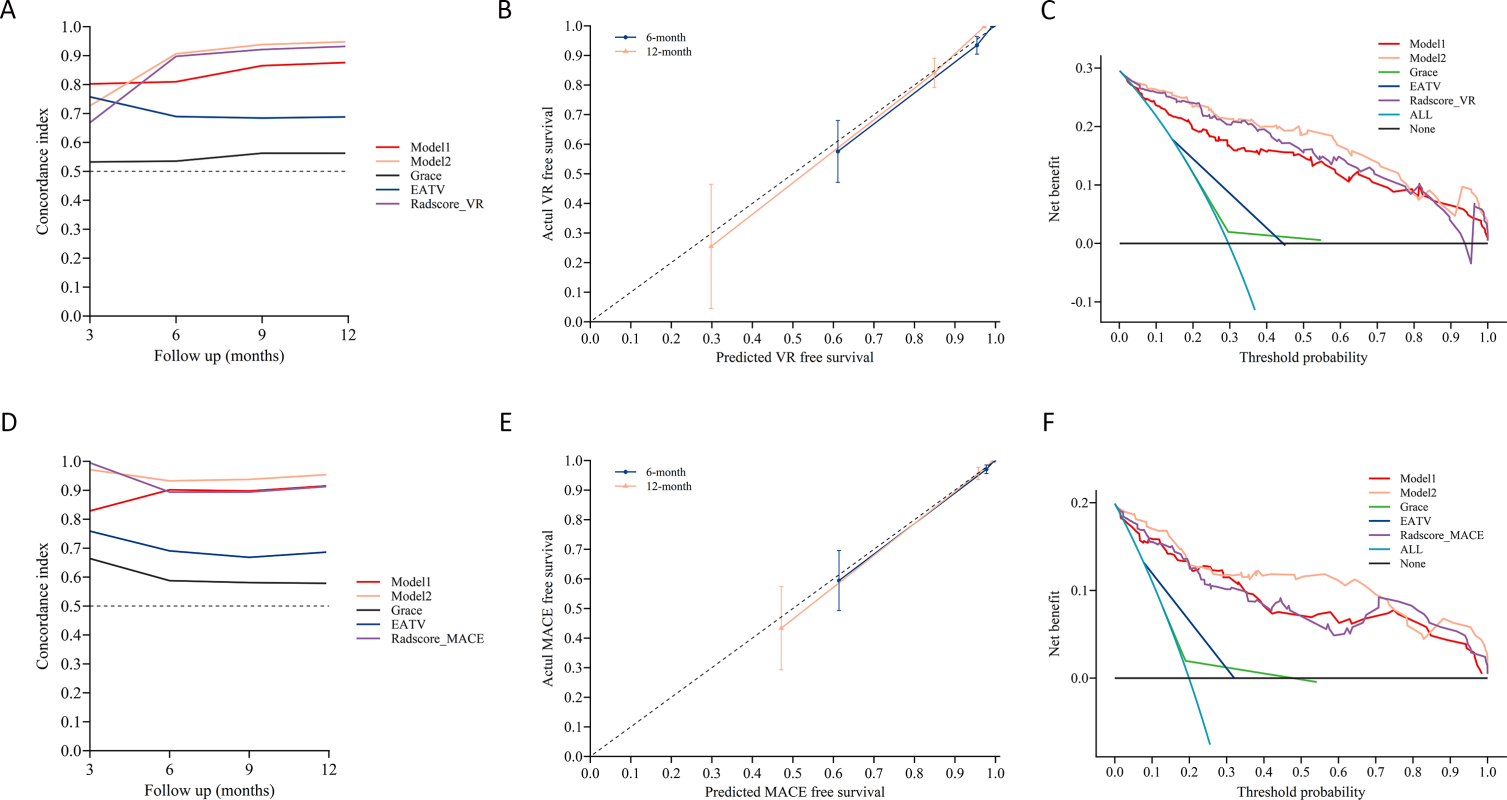
Performance evaluation of model 1 and model 2 for predicting ventricular remodeling (VR) and major adverse cardiovascular events (MACE). **(A, D)** time-dependent concordance index (C-index) comparisons among model 1, model 2, and the GRACE score for VR **(A)** and MACE **(D)** prediction, demonstrating the superior discriminatory ability of model 2 over time. **(B, E)** calibration curve for model 2 predicting VR **(B)** and MACE **(E)**. **(C, F)** decision curve analysis to compare model 1, model 2, and the GRACE score for predicting VR **(C)** and MACE **(F)**.

The time-dependent C-index analysis further confirmed the superior performance of Model 2 over Model 1 and the traditional GRACE score across the entire follow-up period (**Figures 7A, D**). Specifically, Model 2-VR consistently maintained the highest predictive accuracy and its C-index stabilized around 0.90, which outperformed Model 1-VR and GRACE score. Likewise, Model 2-MACE also demonstrated stable predictive superiority throughout the 12-month follow-up, and the C-index it achieved was consistently higher than Model 1-MACE and GRACE score.

Calibration curves analysis showed that there was a good agreement between predicted and observed outcomes (**Figure 7B**) and Model 2-MACE (**Figure 7E**), highlighting their accuracy in risk estimation. DCA revealed that Model 2 provided the highest clinical net benefit over a wide range of threshold probabilities for predicting both VR (**Figure 7C**) and MACE (**Figure 7F**), further supporting its clinical applicability and value.

### 3.6 Internal validation of model 2 for predicting VR and MACE

**Figure 8:**
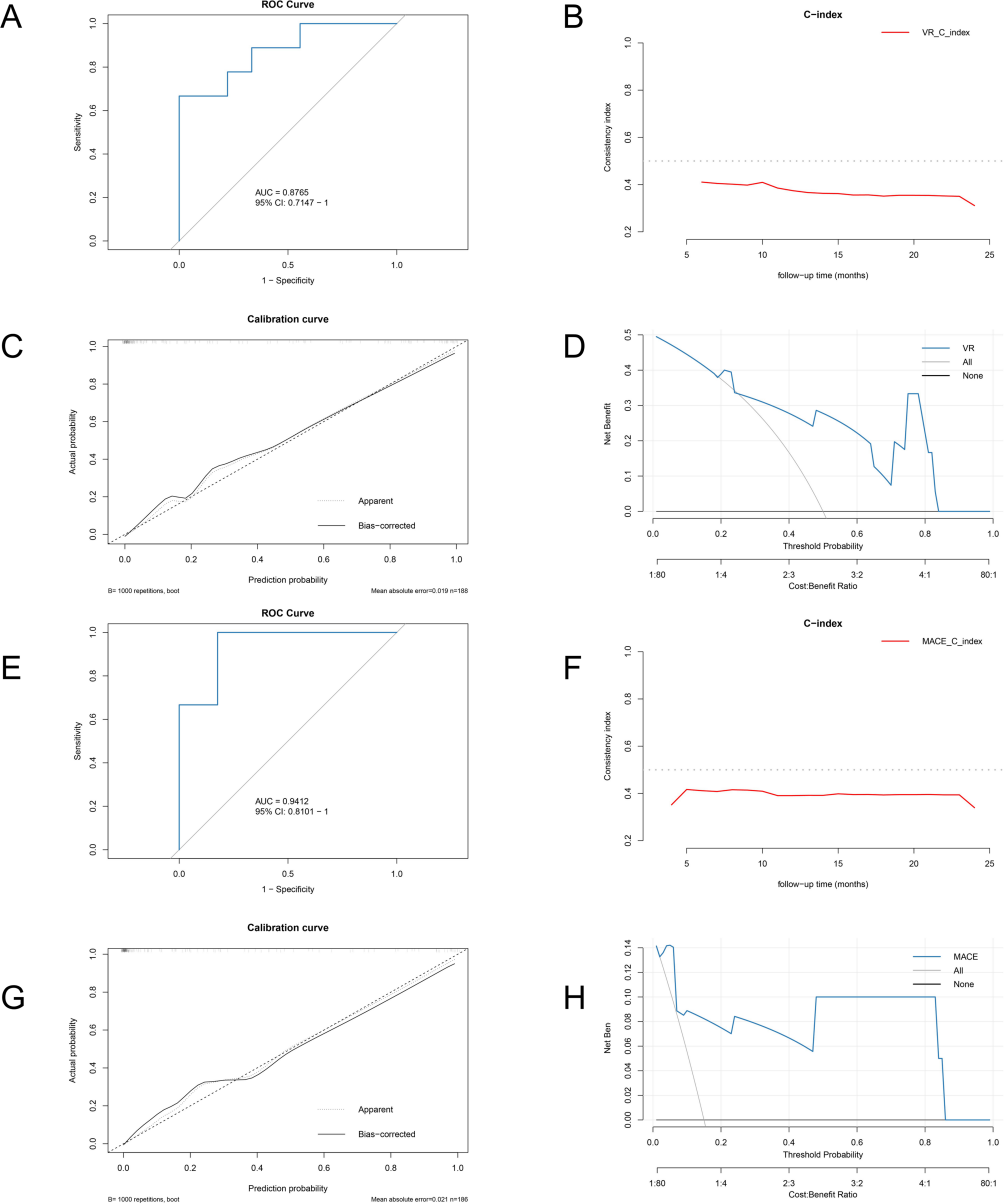
Internal validation of model 2 for predicting VR and MACE. **(A)** ROC curve of model 2 for VR prediction; **(B)** time-dependent C-index of model 2 for VR; **(C)** Calibration curve for VR; **(D)** DCA for VR; **(E)** ROC curve of model 2 for MACE prediction; **(F)** time-dependent C-index of model 2 for MACE; **(G)** calibration curve for MACE; **(H)** DCA for MACE.

To evaluate the robustness and clinical applicability of Model 2, we applied bootstrap resampling (1,000 iterations) for internal validation, and derived model performance metrics from the resampling distributions (**Figure 8**). In terms of VR prediction, the model yielded a favorable discriminatory ability with an AUC of 0.877 (95% CI: 0.715–1.000),and its time-dependent C-index was consistently above 0.750 during follow-up. The calibration curve demonstrated that there was a good agreement between predicted and observed VR probabilities, and DCA indicated that the model had a substantial net benefit within a wide range of threshold probabilities.

Similarly, for MACE prediction, Model 2 also had a strong performance with an AUC of 0.9412 (95% CI: 0.8101–1.000), and its C-index was stable during the follow-up period. There was a good agreement between predicted and observed outcomes based on Calibration analysis, and DCA indicated meaningful clinical net benefit across a range of threshold probabilities. The results above supported the notion that Model 2 has validity and potential utility for individualized risk assessment in AMI patients.

### 3.7 Mechanistic validation

#### 3.7.1 Expansion is associated with aggravated cardiac dysfunction and injury in HFD + ISO-treated rats

**Figure 9:**
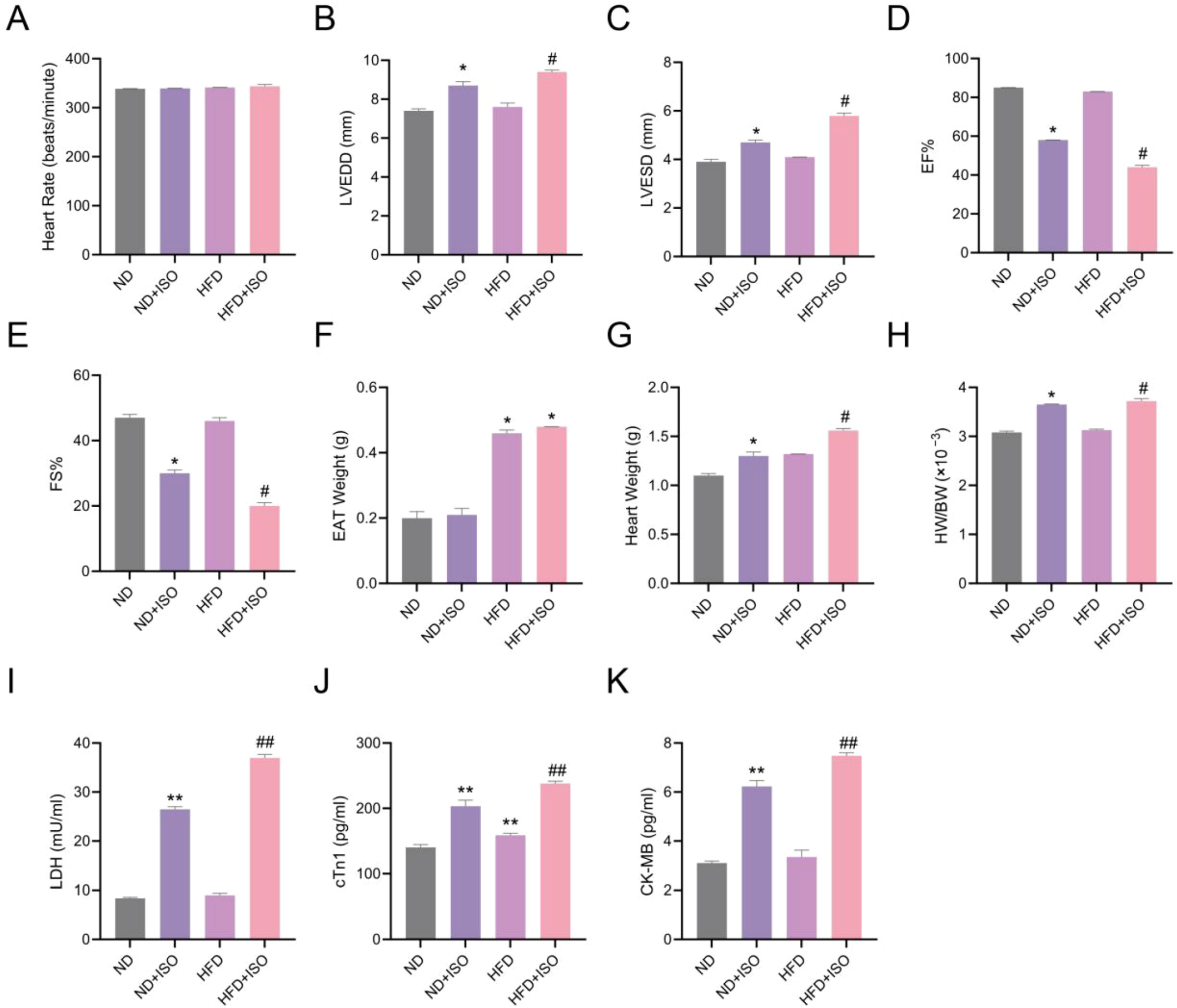
EAT exacerbates cardiac injury and dysfunction in HFD + ISO-treated rats. **(A–E)**. Echocardiographic analysis of heart rate, LVEDD, LVESD, EF, and FS. **(F)** EAT weight. **(G)** Heart weight. **(H)** Heart weight-to-body weight ratio (HW/BW). **(I–K)**. Serum levels of lactate dehydrogenase (LDH), cardiac troponin I (cTnI), and creatine kinase-MB (CK-MB). Data are presented as mean ± SD (n=9). Statistical analysis was performed using one-way ANOVA with Bonferroni *post hoc* test. *P < 0.05, **P < 0.01 vs. ND group; #P < 0.05, ##P < 0.01 vs. HFD group.

To evaluate the association between EAT expansion and cardiac function, a rat model combining HFD and ISO injection was established. Echocardiographic analysis showed that ISO administration significantly increased LVEDD and LVESD, and reduced EF and FS compared to the ND group (P < 0.05). These impairments were further exacerbated in HFD+ISO rats (P < 0.05), indicating a synergistic detrimental effect of metabolic stress and sympathetic stimulation on cardiac function (**Figures 9A–E**).

EAT weight was significantly increased in the HFD and HFD+ISO groups, with the latter showing the greatest increase (**Figure 9F**). HW/BW also peaked in HFD+ISO rat (**Figures 9G, H**). Serum levels of LDH, cTnI, and CK-MB were elevated following ISO treatment and were further increased by HFD preconditioning (P < 0.01) (**Figures 9I–K**), indicating that myocardial injury was more severe under conditions of EAT expansion.

#### 3.7.2 EAT exacerbates myocardial structural injury and fibrosis

**Figure 10:**
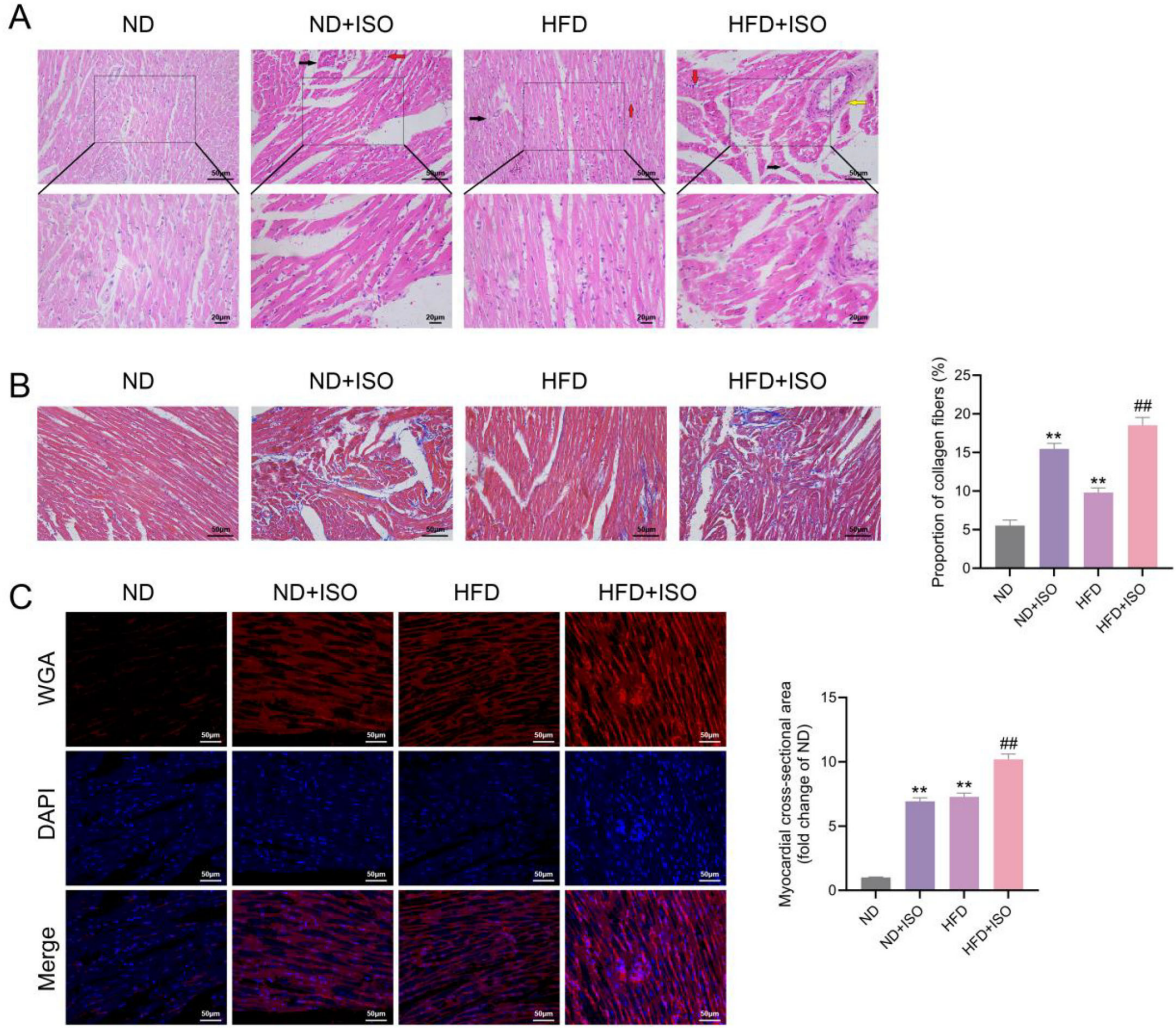
EAT aggravates myocardial injury and remodeling in HFD+ISO-treated rats. **(A)** HE staining showing myocardial morphology across ND, ND+ISO, HFD, and HFD+ISO groups. **(B)** Masson staining for interstitial collagen deposition with quantitative analysis of fibrosis area. **(C)** WGA staining showing cardiomyocyte membranes (red) and nuclei (DAPI, blue); scale bars = 50 μm. Data are presented as mean ± SD (n=9). Statistical analysis was performed using one-way ANOVA with Bonferroni *post hoc* test. **P < 0.01 vs. ND group; ##P < 0.001 vs. HFD group.

Histopathological staining revealed that myocardial injury was markedly aggravated in rats receiving both HFD and ISO. HE staining showed that while ISO or HFD alone induced moderate cardiomyocyte disarray and nuclear pyknosis, the HFD+ISO group exhibited extensive myocardial fiber disruption, edema, and inflammatory infiltration (**Figure 10A**). Masson’s trichrome staining showed a stepwise increase in interstitial collagen deposition from ND + ISO to HFD, reaching the highest collagen volume fraction in HFD + ISO rats (P < 0.01), indicating enhanced extracellular matrix remodeling under combined stress(**Figure 10B**). WGA staining of cross-sectional cardiomyocyte membranes further confirmed myocardial hypertrophy (**Figure 10C**). Cardiomyocyte cross-sectional area was significantly increased in the ISO and HFD groups, with the most pronounced enlargement observed in HFD+ISO rats (P < 0.01).

#### 3.7.3 EAT-derived factors exacerbate hypoxia/reoxygenation-induced cardiomyocyte injury

**Figure 11:**
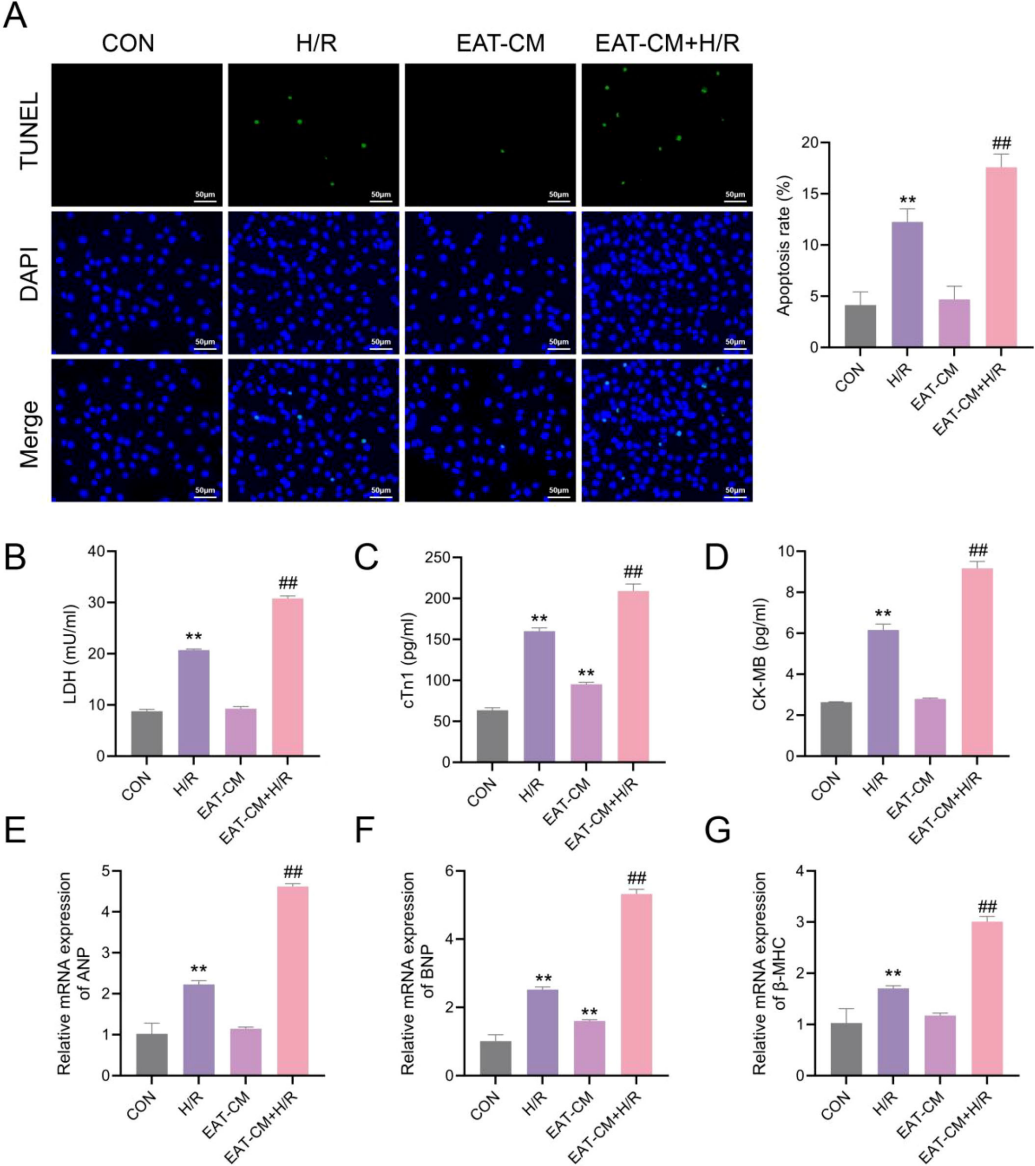
EAT enhances H9c2 cardiomyocyte injury and apoptosis under H/R conditions. **(A)**. TUNEL staining (green) showing apoptotic nuclei, with DAPI counterstaining (blue); Scale bars = 50 μm. **(B–D)** Levels of LDH, cTnI, and CK-MB levels in the culture supernatants. **(E–G)** Relative mRNA expression of ANP, BNP, and β-MHC detected by qRT-PCR. Data are presented as mean ± SD (n=3). Statistical analysis was performed using one-way ANOVA with Bonferroni *post hoc* test. **P < 0.01 vs. CON group; ##P < 0.001 vs. H/R group.

To investigate the paracrine effects of EAT on cardiomyocyte injury, H9c2 cells were exposed to EAT-CM and H/R. TUNEL staining showed minimal apoptosis in control and EAT-CM-only groups, while a significant increase in apoptotic nuclei was observed in the H/R group. Notably, the EAT-CM+H/R group exhibited the highest apoptotic index, suggesting that EAT-derived factors aggravate hypoxia-induced cardiomyocyte apoptosis (**Figure 11A**).

Consistent with these findings, levels of LDH, cTnI, and CK-MB in the culture supernatant were markedly elevated following H/R, and further increased in the EAT-CM+H/R group (P < 0.01) (**Figures 11B–D**), indicating aggravated cell membrane damage. Furthermore, qRT-PCR revealed that the expression of cardiac stress and hypertrophy markers ANP, BNP, and β-MHC were significantly upregulated by H/R stimulation, with the highest expression levels observed in the EAT-CM+H/R group (P < 0.01) (**Figures 11E–G**).

These results demonstrated that EAT-derived paracrine factors amplified cardiomyocyte injury and stress responses under ischemic conditions.

## 4 Discussion

This study aimed to develop and validate radiomics-based predictive models integrating epicardial adipose tissue (EAT) features and clinical parameters for predicting ventricular remodeling (VR) and major adverse cardiovascular events (MACE) after acute myocardial infarction. The most important finding of this study is that the radiomics-integrated model (Model 2) demonstrated superior predictive performance compared with Model 1 and the GRACE score. In parallel, *in vivo* and *in vitro* experiments provided mechanistic evidence that EAT expansion is closely associated with aggravated myocardial injury, fibrosis, and hypertrophy under metabolic and ischemic stress. This integrative approach links imaging biomarkers to biological mechanisms, supporting the dual role of EAT as both a predictive indicator and a pathogenic mediator.

Our findings confirm the prognostic relevance of EAT in AMI outcomes, consistent with previous studies linking EAT burden to ventricular dysfunction and adverse events (25, 26).

This study extends conventional volume-based assessment by incorporating radiomics to extract quantitative features of texture, shape, and intensity heterogeneity from CT images. High-ranking features such as GLCM-IDMN, reflecting spatial uniformity, may capture underlying biological changes including inflammation or fibrosis. This may explain why the Rad-score model outperformed the volume-based model in predicting VR and MACE (27, 28).

The experimental validation provided direct biological plausibility for the imaging findings. In the HFD+ISO rat model, EAT expansion coincided with more severe systolic dysfunction, increased myocardial fibrosis, and cardiomyocyte hypertrophy compared to ISO alone. These effects support that metabolically active EAT secretes bioactive molecules, including pro-inflammatory cytokines such as TNF-α, IL-6, and MCP-1, adipokines, free fatty acids, and profibrotic mediators such as TGF-β, which act on the adjacent myocardium and exacerbate inflammation, oxidative stress, and extracellular matrix remodeling (29, 30). The *in vitro* H9c2 experiments further supported a paracrine injury mechanism: EAT-conditioned medium amplified hypoxia/reoxygenation-induced apoptosis, increased LDH, cTnI, and CK-MB release, and upregulated fetal cardiac genes (ANP, BNP, β-MHC). Together, these findings suggest that predictive radiomics features may reflect biologically active EAT phenotypes contributing to myocardial injury and maladaptive remodeling.

From a clinical standpoint, incorporating EAT radiomics features into prediction models enables earlier identification of AMI patients at high risk for adverse remodeling, even when traditional risk factors suggest lower risk. Given the routine use of cardiac CT in coronary evaluation, EAT quantification and radiomics analysis could be integrated into standard imaging workflows with minimal additional cost or time. Moreover, our mechanistic results raise the possibility that EAT is not only a marker but also a modifiable target. Pharmacological interventions such as SGLT2 inhibitors and GLP-1 receptor agonists, as well as lifestyle modifications, have been shown to reduce EAT volume and inflammation (31, 32), offering potential for targeted therapy in patients identified as high risk by EAT Rad-score.

The association between greater EAT burden and adverse post-AMI outcomes is consistent with previous studies (25, 33), supporting the robustness of our findings across populations and imaging modalities. While earlier studies mainly focused on EAT volume, our work incorporated advanced radiomics modeling and mechanistic experiments, thereby linking quantitative imaging phenotypes with underlying biological processes. This integrated approach not only confirmed known clinical associations but also suggested that radiomics features predictive of adverse outcomes may correspond to metabolically and structurally active EAT phenotypes. By combining patient-level prognostic modeling with experimental validation, our study provides a novel translational framework for identifying high-risk individuals and for exploring targeted therapeutic strategies.

Several limitations should be acknowledged. First, this was a single-center study with a modest sample size, which may limit generalizability; potential confounding from post-AMI treatments and unassessed lifestyle factors cannot be excluded; thus, external validation is warranted. Second, although the *in vivo* and *in vitro* experiments provided mechanistic insights, they cannot fully recapitulate the complex EAT–myocardium interactions in human AMI, and the experimental disease models were limited to high-fat feeding and β-adrenergic stimulation without inclusion of other comorbid conditions such as diabetes or chronic inflammation, and the combined HFD and ISO design limits causal inference regarding EAT. Third, reproducibility of radiomics features may be affected by variability in CT acquisition and segmentation, underscoring the need for standardized protocols and cross-platform harmonization. Fourth, the imaging biomarkers were not directly validated against histopathological measurements of EAT activity in human samples, which would enhance biological interpretability. Fifth, while SHAP analysis improved model interpretability, its clinical applicability requires further development into user-friendly decision-support tools. Finally, this study did not assess whether targeted reduction of EAT burden or modification of its biological activity can mitigate the risk of VR and MACE, which should be a focus of future interventional research.

## 5 Conclusion

This study demonstrated that integrating EAT radiomics features with clinical parameters significantly improves the prediction of ventricular remodeling and major adverse cardiovascular events after acute myocardial infarction.

From a clinical and public health perspective, incorporating EAT-derived radiomics into routine risk assessment may enhance early identification of high-risk patients and support more precise stratification beyond conventional indicators. This approach aligns with current health benchmarks emphasizing personalized medicine and risk-adapted management, potentially facilitating timely interventions, optimizing follow-up strategies, and improving long-term cardiovascular outcomes.

Future studies should focus on external validation in multicenter cohorts and the standardization of radiomics workflows to improve reproducibility. In addition, further mechanistic investigations are warranted to clarify the biological role of EAT in post-AMI remodeling and to explore its potential as a therapeutic target.

## Data Availability

The original contributions presented in the study are included in the article/**Supplementary Material**. Further inquiries can be directed to the corresponding authors.
