# Supplementary material for: Epicardial adipose tissue radiomics predicts VR and MACE after AMI: a prospective cohort study
Source: Front Endocrinol (Lausanne). 2026 May 18;17:1781007. doi: 10.3389/fendo.2026.1781007 (PMC13222965; doi:10.3389/fendo.2026.1781007)
Supplement: Supplementary file 1 [file DataSheet1.docx]

**
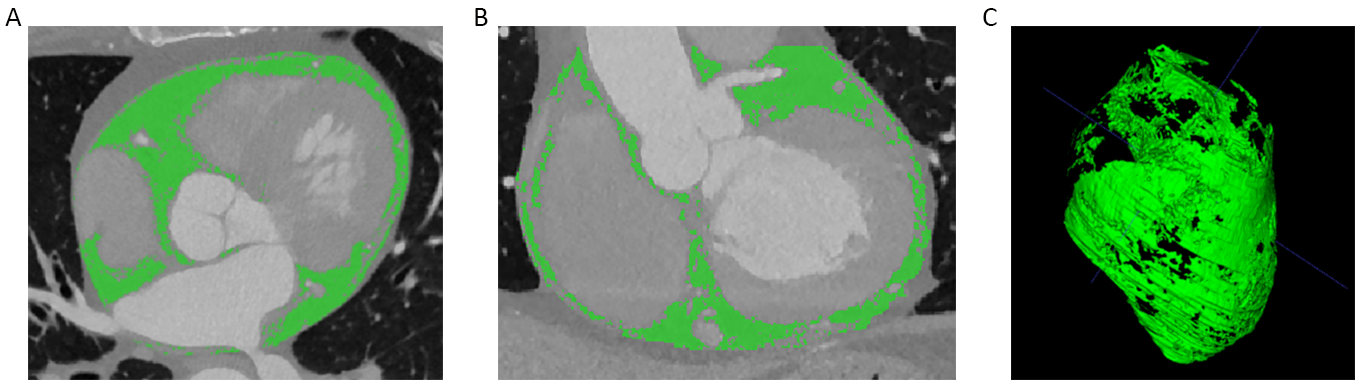
**

**Supplementary Figure 1. Representative examples of epicardial adipose tissue (EAT) segmentation on cardiac computed tomography angiography (CCTA) images.**
(A) Axial view; (B) coronal view; (C) three-dimensional reconstruction of segmented EAT (green) using 3D Slicer software. EAT was defined as adipose tissue within the pericardial contour with attenuation between −200 and +30 Hounsfield units.


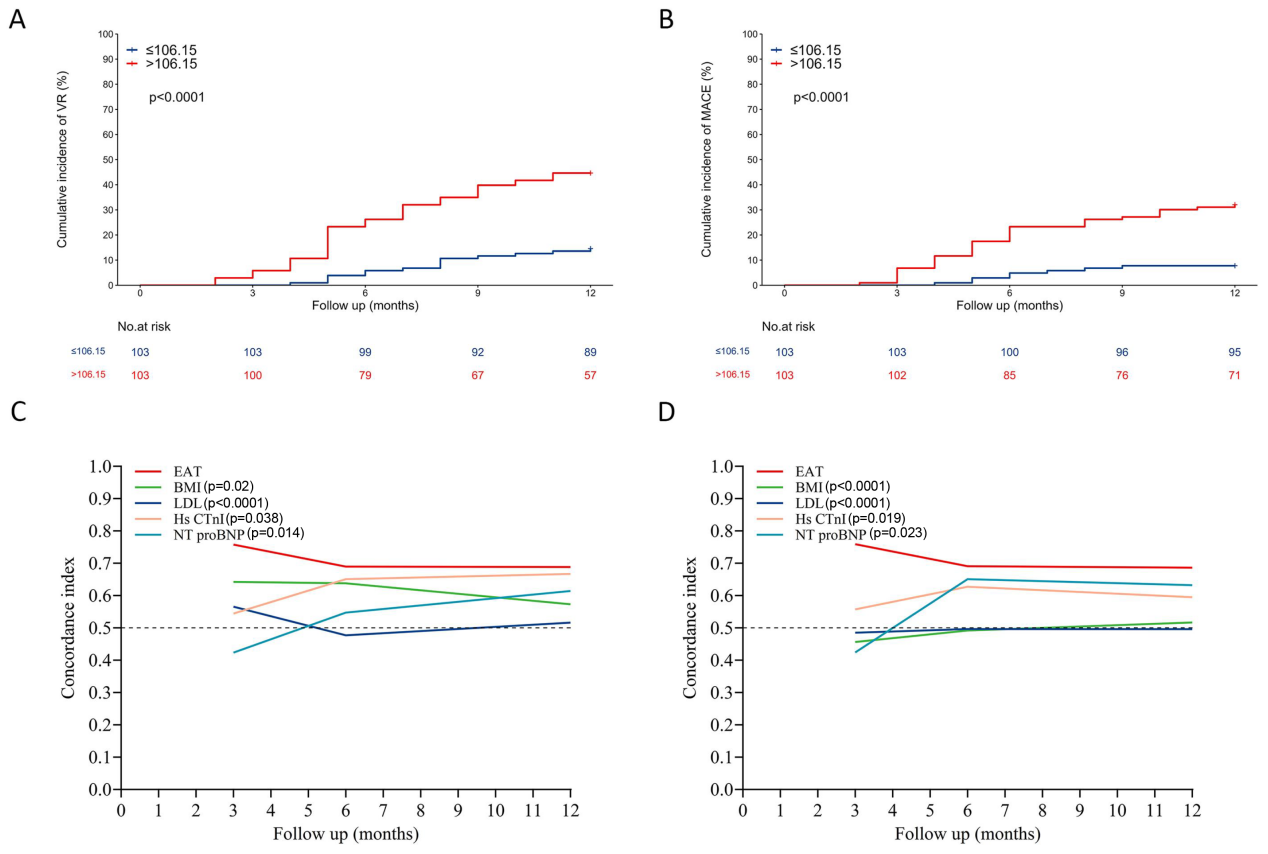


**Supplementary Figure 2. Prognostic value of EAT volume for ventricular remodeling (VR) and major adverse cardiovascular events (MACE) after acute myocardial infarction (AMI).**
(A–B) Kaplan–Meier curves showing cumulative incidence of VR and MACE in patients stratified by the median EAT volume (106.5 cm³). (C–D) Time-dependent concordance index (C-index) analysis comparing EAT volume with other clinical predictors (BMI, LDL, Hs-cTnI, and NT-proBNP).


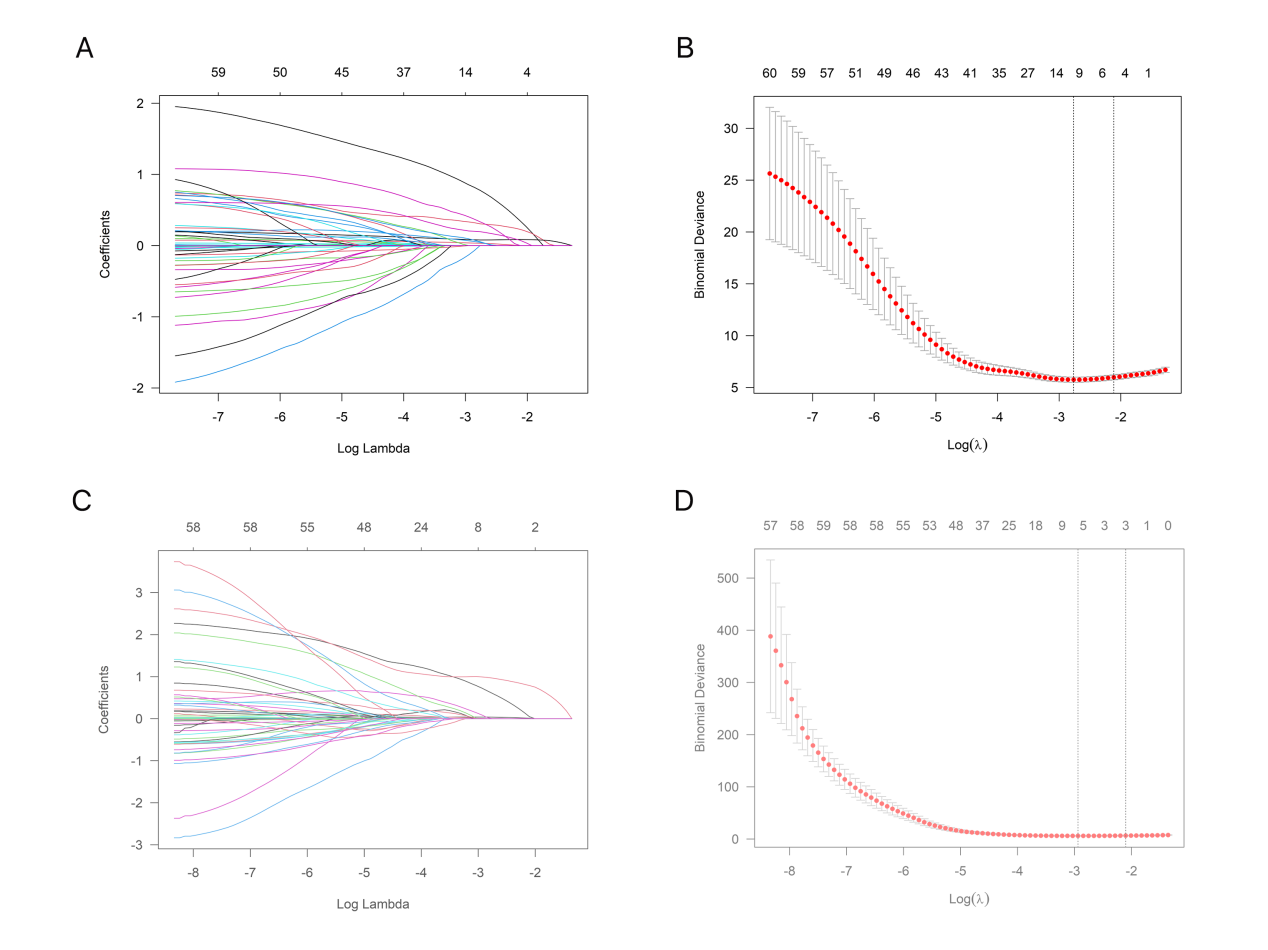


**Supplementary Figure 3. Least absolute shrinkage and selection operator (LASSO) regression for feature selection in VR and MACE prediction models.**
(A–B) LASSO coefficient profiles and optimal tuning parameter (λ) selection for VR. (C–D) LASSO coefficient profiles and optimal tuning parameter (λ) selection for MACE. The vertical dashed line indicates the λ value corresponding to the minimum cross-validation error.


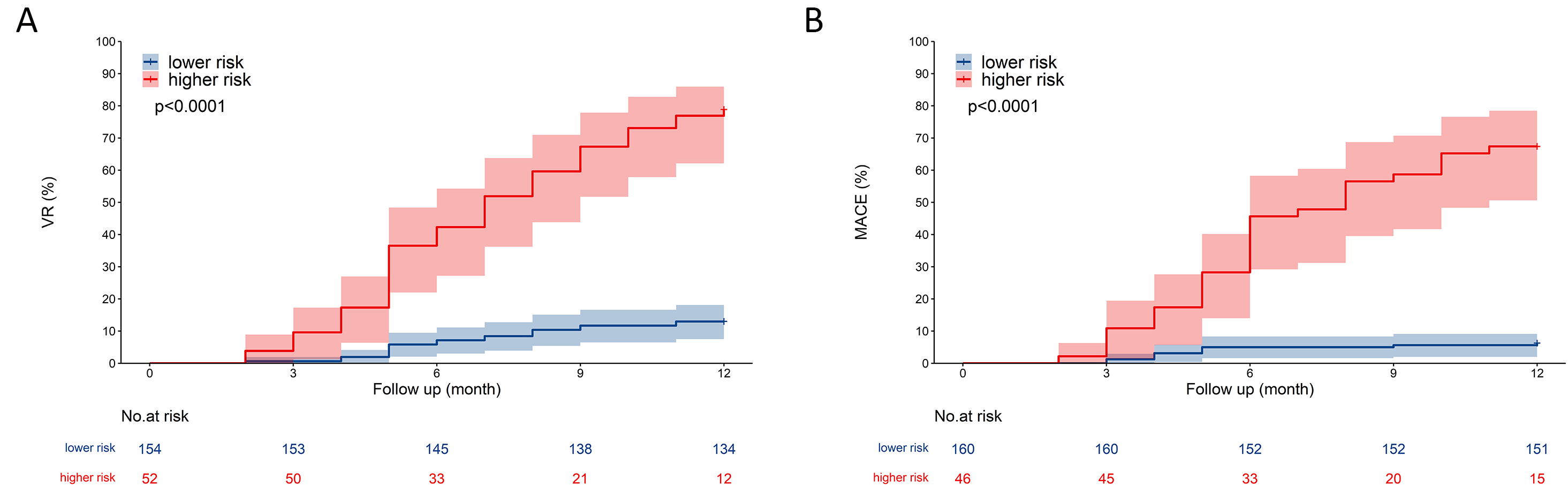


**Supplementary Figure 4. Kaplan–Meier survival curves for VR (A) and MACE (B) based on risk stratification using Model 1 nomograms.** Higher-risk groups exhibited significantly greater cumulative event rates than lower-risk groups (log-rank test, p < 0.0001).


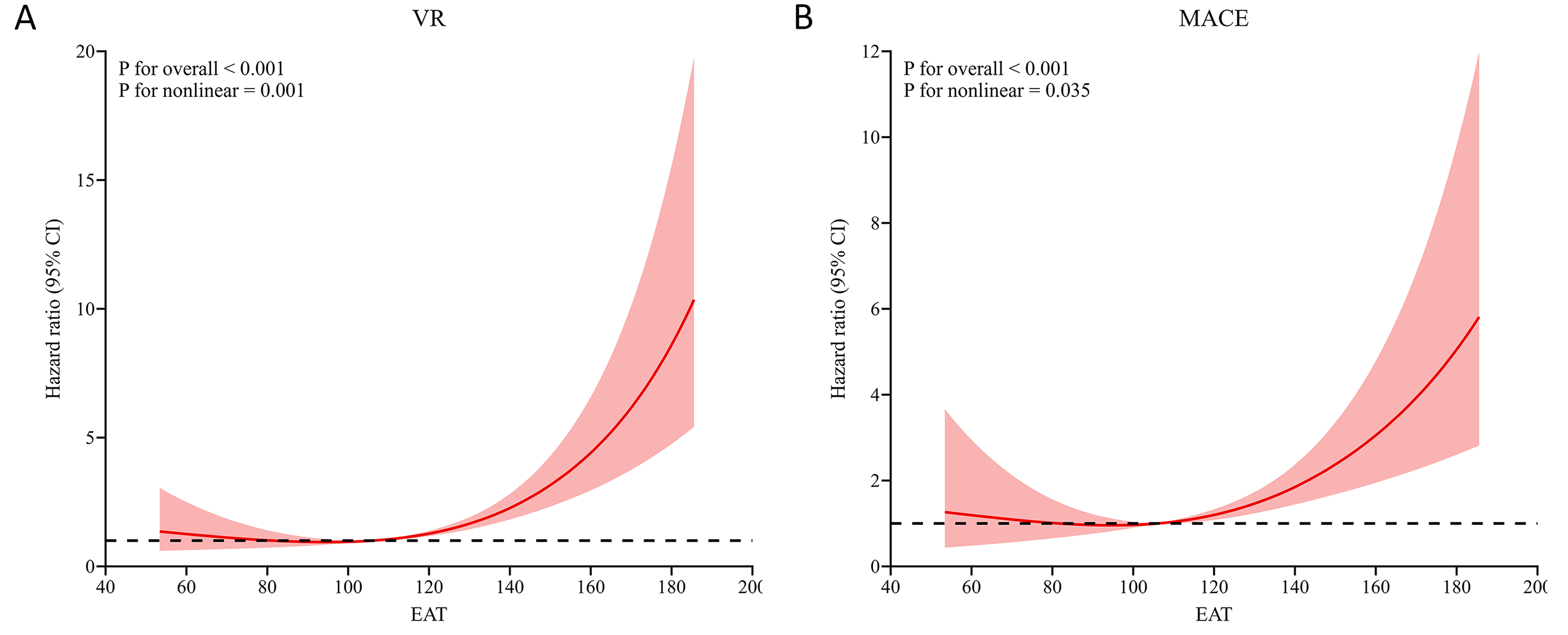


**Supplementary Figure 5. Restricted cubic spline analysis of the association between EAT volume and the risk of (A) VR and (B) MACE.** A nonlinear relationship was observed with an inflection point around 106.5 cm³, beyond which risk increased sharply. Shaded areas represent 95% confidence intervals.


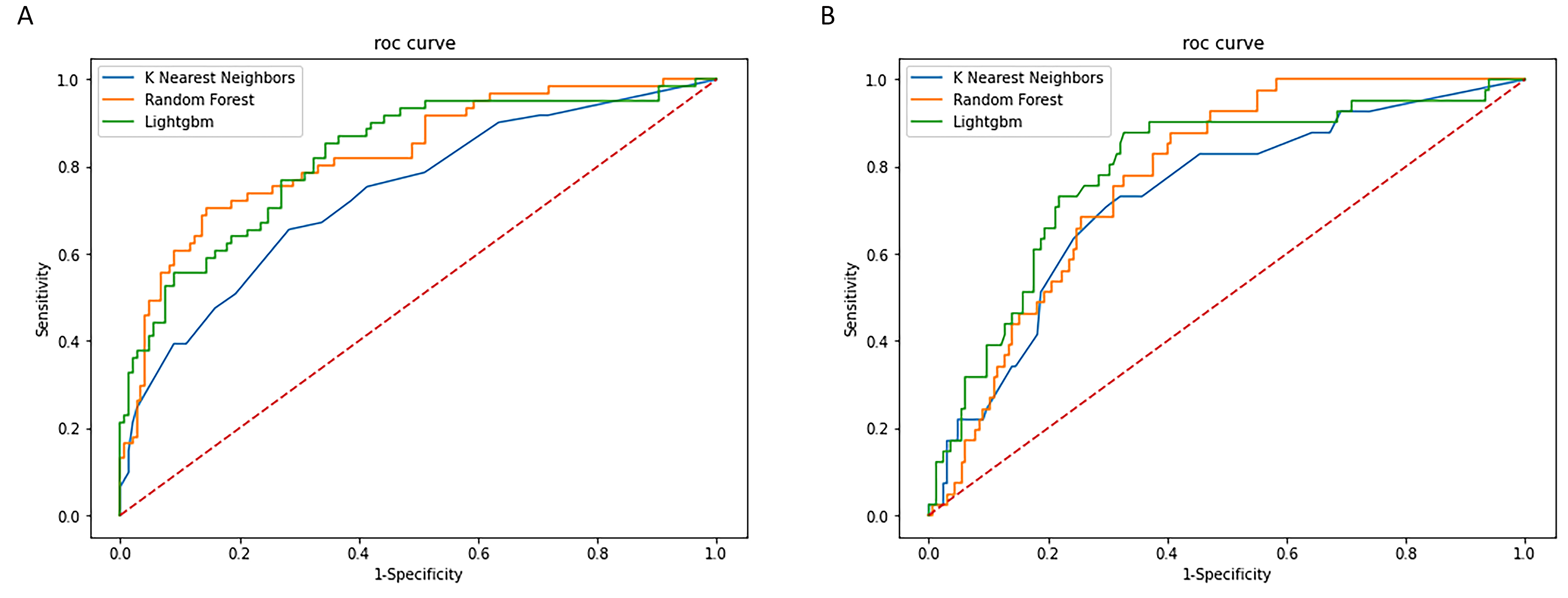


**Supplementary Figure 6. Comparison of machine learning algorithms for predicting (A) VR and (B) MACE based on selected radiomics features.** Receiver operating characteristic (ROC) curves show that the Random Forest algorithm achieved the highest AUC for VR prediction, while the LightGBM algorithm performed best for MACE prediction.
