# Supplementary material for: Epicardial adipose tissue radiomics predicts VR and MACE after AMI: a prospective cohort study
Source: Front Endocrinol (Lausanne). 2026 May 18;17:1781007. doi: 10.3389/fendo.2026.1781007 (PMC13222965; doi:10.3389/fendo.2026.1781007)
Supplement: Supplementary file 2 [file DataSheet2.doc]

**Supplementary Table 1 List of variable assignment**

| Variables | Assignment | Variables | Assignment |
| --- | --- | --- | --- |
| Gender | Male = 0; Female = 1 | Alcohol consumption | None = 0; Yes = 1 |
| Smoking history | None = 0; Yes = 1 | Hypertension history | None = 0; Yes = 1 |
| Diabetes history | None = 0; Yes = 1 | Family history of coronary heart disease | None = 0; Yes = 1 |
| Atrial fibrillation at admission | None = 0; Yes = 1 | II^。^or above AVB | None = 0; Yes = 1 |
| LAD occlusion site | Near segment= 0 Middle segment= 1  distal segment=2 | Intraoperative No-reflow/Slow-reflow | None = 0; Yes = 1 |
| Respiratory and cardiovascular comorbidities | None = 0; Yes = 1 | Ventricular arrhythmia at admission | None=0; Frequent ventricular premature beats or short-duration ventricular tachycardia=1; sustained ventricular tachycardia or ventricular fibrillation=2 |
| Intraoperative hypotension | None = 0; Yes = 1 | Intraoperative ventricular arrhythmia | None=0; Frequent ventricular premature beats or short-duration ventricular tachycardia=1; persistent ventricular tachycardia or ventricular fibrillation=2 |
| Postoperative ACEI/ARB/ARNI use | None = 0; Yes = 1 | Postoperative β-blocker use | None = 0; Yes = 1 |
| Postoperative SGLT2i use | None = 0; Yes = 1 | Postoperative MRA use | None = 0; Yes = 1 |
| Killip classification | Grade I=0; Grade II=1; Grade III=2; Grade IV=3 | | |
| LVDF | No diastolic dysfunction = 0; Level I = 1; Level II = 2; Level III = 3 | | |
| Number of diseased branches | 1 diseased branch= 0; 2 diseased branch=1; 3 diseased branch=2 | | |
| TIMI flow classification | Level 3 = 0; Level 2 = 1; Level 1 = 2; Level 0 = 3 | | |
| Age | ≤50 years=0 ; 51-69 years=1; ≥70 years=2 | | |
| BMI | <18.5kg/m^2^=0; 18.5-24.9kg/m^2^=1; 25-29.9kg/m^2^=2; ≥30kg/m^2^=3 | | |
| MAP at admission | ≥65mmHg=0; <65mmHg=1 | | |
| Heart rate at admission | ≤100 beats per minute=0; >100 beats per minute=1 | | |
| D2W time | ≤90 minutes=0; >90 minutes=1 | | |
| NT-pro BNP | <250pg/ml=0 ; 250-450pg/ml=1; 450-1700pg/ml=2; 1700-4200pg/ml=3; >4200pg/ml=4 | | |
| Hs-cTnI | <130.15ng/L=0; 130.15-1942.4ng/L=1; 1942.4-24058.47ng/L=2; >24058.47ng/L=3 | | |
| MYO | <30=0 ng/ml; 30-51.4ng/ml=1; 51.4-198.5ng/ml=2; >198.5ng/ml=3 | | |
| CK-MB | <9.25ng/ml=0; 9.25-17.17ng/ml=1; 17.17-43ng/ml=2; >43ng/ml=3 | | |
| Hs-CRP | <1.0mg/L=0; 1.0-3.0mg/L=1; >3.0mg/L=2 | | |
| LVEF | ≥50%=0 ; 40%-49%=1: <40%=2 | | |

**Supplementary Table 2 Cox proportional hazard regression analysis for identification of significant clinical variables for VR and MACE**

| Variables for VR | HR (95% CI) | P |
| --- | --- | --- |
| EAT volume (>106.15 vs ≤106.15cm^3^) | 4.859 (2.553, 9.247) | <0.001 |
| LVEF (1 vs 0) | 2.472 (1.077, 5.675) | 0.033 |
| LVEF (2 vs 0) | 3.285 (1.640, 6.583) | 0.001 |
| DETERMINE Score | 1.105 (1.035, 1.179) | 0.003 |
| hs-CTnI (1 vs 0) | 2.094 (0.804, 5.453) | 0.130 |
| hs-CTnI (2 vs 0) | 2.566 (1.103, 5.967) | 0.029 |
| hs-CTnI (3 vs 0) | 2.708 (1.068, 6.864) | 0.036 |
| No reflow/Slow blood flow(1 vs 0) | 2.258 (1.311, 3.887) | 0.003 |
| Variables for MACE | HR (95% CI) | P |
| EAT volume (>106.15 vs ≤106.15cm^3^) | 5.165 (2.325, 11.474) | <0.001 |
| LVEF (1 vs 0) | 4.323 (1.769, 10.567) | 0.001 |
| LVEF (2 vs 0) | 14.430 (6.519, 31.944) | <0.001 |
| Determine Score | 1.087 (1.004, 1.177) | 0.039 |
| No reflow/Slow blood flow(1 vs 0) | 2.247 (1.168, 4.323) | 0.015 |

**Supplementary Table 3 Cox proportional hazard regression analysis for identification of significant clinical variables and Radscore for VR and MACE**

| Variables for ventricular remodel | HR (95% CI) | P |
| --- | --- | --- |
| Radscore | 3.430 (2.601, 4.522) | <0.001 |
| LVWDI | 1.071 (1.005, 1.142) | 0.034 |
| Killip Classification (II vs I) | 1.228 (0.629, 2.399) | 0.548 |
| Killip Classification (III vs I) | 1.728 (0.877, 3.407) | 0.114 |
| Killip Classification (IV vs I) | 4.702 (1.590, 13.902) | 0.005 |
| Variables for MACE | HR (95% CI) | P |
| Radscore | 3.342 (2.371, 4.713) | <0.001 |
| LVEF (1 vs 0) | 2.855 (1.192, 6.841) | 0.019 |
| LVEF (2 vs 0) | 7.444 (3.337, 16.606) | <0.001 |
| No reflow/Slow blood flow (1 vs 0) | 2.315 (1.217, 4.405) | 0.005 |

**Supplementary Table 4. Primer sequences used in this study.**

| GeneSequence (5'→3')*ANP*Forward: TAATGGGCTCCTTCTCCATCReverse: TTATCTTCGGTACCGGAAGC*BNP*Forward: TAATGGATCTCCAGAAGGTGCReverse: TTCTTTTGTAGGGCCTTGGTC*β-MHC*Forward: CAAATCATCCAAGCCAACCCReverse: CTCTTTCTGCTTTCAGCTGG*GAPDH*Forward: ACTCCCTCAAGATTGTCAGCReverse: AGTTGCTGTTGAAGTCACAGG |  | |
| --- | --- | --- |
|  | |  |
|  | |  |
|  | |  |
|  | |  |
